# Triplet Emitting C^N^C Cyclometalated Dibenzo[c,h]Acridine Pt(II) Complexes

**DOI:** 10.3390/molecules27228054

**Published:** 2022-11-19

**Authors:** Joshua Friedel, Maren Krause, Rose Jordan, Iván Maisuls, Dana Brünink, Dominik Schwab, Nikos L. Doltsinis, Cristian A. Strassert, Axel Klein

**Affiliations:** 1 Universität zu Köln, Fakultät für Mathematik und Naturwissenschaften, Department für Chemie, Institut für Anorganische Chemie, Greinstraße 6, 50939 Köln, Germany; 2 Institut für Anorganische und Analytische Chemie, CiMIC, CeNTech, Westfälische Wilhelms-Universität Münster, Heisenbergstraße 11, 48149 Münster, Germany; 3 Institut für Festkörpertheorie and Center for Multiscale Theory and Computation, Westfälische Wilhelms-Universität Münster, Wilhelm-Klemm-Straße 10, 48149 Münster, Germany

**Keywords:** platinum, cyclometalating CNC ligands, time-resolved photoluminescence spectroscopy, DFT calculations, electrochemistry

## Abstract

In a series of Pt(II) complexes [Pt(dba)(L)] containing the very rigid, dianionic, *bis*-cyclometalating, tridentate C^N^C^2−^ heterocyclic ligand dba^2–^ (H_2_dba = dibenzo[c,h]acridine), the coligand (ancillary ligand) L = dmso, PPh_3_, CN*t*Bu and Me_2_Imd (*N*,*N*’-dimethylimidazolydene) was varied in order to improve its luminescence properties. Beginning with the previously reported dmso complex, we synthesized the PPh_3_, CN*t*Bu and Me_2_Imd derivatives and characterized them by elemental analysis, ^1^H (and ^31^P) NMR spectroscopy and MS. Cyclic voltammetry showed partially reversible reduction waves ranging between −1.89 and −2.10 V and increasing along the series Me_2_Imd < dmso ≈ PPh_3_ < CN*t*Bu. With irreversible oxidation waves ranging between 0.55 (L = Me_2_Imd) and 1.00 V (dmso), the electrochemical gaps range between 2.65 and 2.91 eV while increasing along the series Me_2_Imd < CN*t*Bu < PPh_3_ < dmso. All four complexes show in part vibrationally structured long-wavelength absorption bands peaking at around 530 nm. TD-DFT calculated spectra agree quite well with the experimental spectra, with only a slight redshift. The photoluminescence spectra of all four compounds are very similar. In fluid solution at 298 K, they show broad, only partially structured bands, with maxima at around 590 nm, while in frozen glassy matrices at 77 K, slightly blue-shifted (~580 nm) bands with clear vibronic progressions were found. The photoluminescence quantum yields *Φ*_L_ ranged between 0.04 and 0.24, at 298 K, and between 0.80 and 0.90 at 77 K. The lifetimes *τ* at 298 K ranged between 60 and 14040 ns in Ar-purged solutions and increased from 17 to 43 µs at 77 K. The TD-DFT calculated emission spectra are in excellent agreement with the experimental findings. In terms of high *Φ*_L_ and long *τ*, the dmso and PPh_3_ complexes outperform the CN*t*Bu and Me_2_Imd derivatives. This is remarkable in view of the higher ligand strength of Me_2_Imd, compared with all other coligands, as concluded from the electrochemical data.

## 1. Introduction

Studies on luminescent transition metal complexes are motivated by their wide range of potential applications, in fields such as photocatalysis [1,2], sensing [3,4,5], optoelectronic devices [5,6,7,8,9,10,11], and biomedicine [4,5,12,13,14,15]. Phosphorescent metal complexes are of particular interest in the field of OLED (Organic Light Emitting Diodes) applications, due to the ability of these materials to harvest all generated excitons in electroluminescent devices, by means of efficient spin-orbit coupling (SOC) promoting intersystem crossing and phosphorescence, which is associated with the heavy metal centers [5,7]. Frequently, metal-containing phosphorescent emitters involve Ir(III), Ru(II) or Pt(II) centers [3,4,5,6,7,8,9,10,11,12,13,14,15,16,17,18,19,20,21,22]. While the d^6^ species, such as Ir(III) and Ru(II), often adopt an octahedral coordination environment, metals with a d^8^ electron configuration (such as Pt(II), Pd(II) and Au(III), among others) often display square-planar geometries with open coordination flanks in the axial positions [4,16,17]. These open axial positions can lead to metal-metal (M···M) and/or π-stacking interactions upon aggregation with red-shifted emission from MMLCT states (metal-metal-to-ligand charge transfer, eventually with excimeric M^...^M shortening), as opposed to the LC (ligand-centered) character found in monomeric species [5,11,18,19,20,21,22].

Two main structural features have been discovered to be beneficial for efficient triplet luminescence in Pt(II) complexes. The first is rigid coordination environments, which prevent radiationless decay from the triplet excited states; the second is the use of polydentate heteroaromatic cyclometalated chromophores [5,9,16,17,20,21,22,23,24,25,26,27,28,29,30,31,32,33,34,35,36,37,38,39,40,41,42,43,44,45,46,47,48,49,50,51,52,53,54]. Such ligands energetically disfavor the thermal population of “dark” (dissociative) d-d* excited states, which are responsible for non-radiative decay paths via conical intersections with the ground state, and they also provide multiple options for the character of the excited triplet states (such as the metal(d^8^)-to-ligand(π*) charge transfer (MLCT) configurations, intraligand (π–π*) or charge-transfer excitations involving donor and acceptor parts of the ligand(s) called LL’CT states as well as mixtures thereof) [5,8,10,11,16,17,21]. Thus, a number of Pt(II) complexes containing tri- or tetra-dentate multifunctional (*C*-bonding, *N*-bonding, further substituents) ligands were studied previously [4,5,17,20,21,22,23,24,25,26,27,28,29,30,31,32,33,34,35,36,37,38,39,40,41,42,43]

**Scheme 1:**
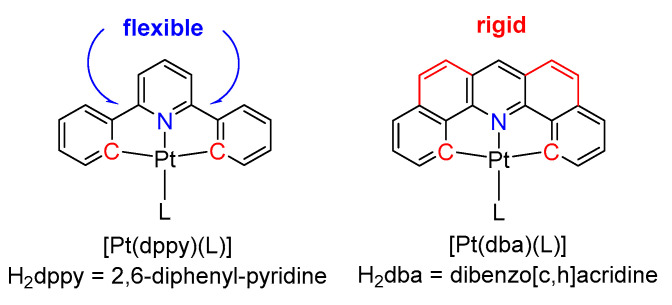
Schematic depiction of C^N^C Pt(II) complexes with the relatively flexible dppy ligand (left) or the rather rigid dba ligand (right).

Amongst the simplest tridentate ligands, variations of the C^N^N, N^C^N and C^N^C cores based on phenyl(*C*) and pyridyl(*N*) donors have been reported with the double cyclometalation C^N^C having no marked benefit over the C^N^N or N^C^N coordination in terms of the efficiency of the triplet photoluminescence [33,45,46,47]. On the other hand, the doubly anionic C^N^C^2−^ ligands allow researchers to choose from a variety of neutral L coligands (ancillary ligands), whereas the C^N^N and N^C^N derivatives require anionic coligands to yield neutral cyclometalated Pt(II) complexes. The parent 2,6-diphenyl-pyridine (dppy) type of C^N^C ligands (see Scheme 1, i.e., the derivatives [Pt(dppy)(L)] (H_2_dppy = 2,6-diphenyl-pyridine, L = dimethyl sulfoxide (dmso), pyridine (Py), 4-*t*Bu-Py, MeCN, PPh_3_, PCy_3_, CNXyl (Xyl = 2,6-xylyl), Me-4,4′-bipyridinium, and the PPh_2_-crown ether-based species) showed no photoluminescence (PL) at ambient *T* in the solution [33,43,45,46]. In contrast to this finding, PL at ambient *T* in the solution was reported for L = pyridine derivatives with pending crown ether moieties [46] On the other hand, the expansion of one phenyl group to naphthyl and the central pyridine to 4-phenyl-pyridine did not yield PL at 298 K in the solution for L = dmso or CNXyl, while the replacement of the peripheral phenyl groups by thiophenyl or *N*-alkyl carbazolyl moieties reached efficient PL intensities under these conditions [47]. In line with the rather poor performance of the simple dppy ligand, very recently, the complexes [Pt(L)(dmso)] with the same type of flexible C^N^C ligands (H_2_L *=* 2,6-di(phenyl)-4-(3,4,5-X-phenyl)-pyridine, X = H or F), were reported as being non-emissive while showing very interesting four-photon absorption (4PA), which can be used for medical diagnosis [48]. A very recent theoretical work on these complexes focussed on the 4PA singlet-singlet excitation, followed by ISC to the triplet states *T*_2_ and *T*_1_ [49].

In contrast to the dppy system, in which the C–C single bonds between the phenyl and the pyridyl groups provides some flexibility for deformations (Scheme 1, left), the condensation of the three aromatic rings in the dba^2−^ (H_2_dba = dibenzo[c,h]acridine) ligand (Scheme 1, right) led to a far more rigid coordination environment. In our previous study, we found markedly increased PL quantum yields *Φ*_L_ for the complex [Pt(dba)(dmso)] (0.23 at 298 K in CH_2_Cl_2_ solution), compared with derivatives of [Pt(dppy)(dmso)] [33] (no emission under these conditions), which is in line with the idea that molecular rigidity helps to reduce or circumvent radiationless decay paths. This makes these Pt dba complexes interesting candidates for various applications.

We recently reported a similar strategy for C^N^N and N^C^N cyclometalated Pt(II) complexes, including the complexes [Pt(naphen)(X)] (Hnaphen = naphtho[1,2-b][1,10]phenanthroline, X = Cl or C≡CPh) with the rigid tridentate C^N^N-coordinating pericyclic naphen ligand, the slightly more flexible tetrahydro-derivative [Pt(thnaphen)(X)] (Hthnaphen = 5,6,8,9-tetrahydro-naphtho[1,2-b][1,10]phenanthroline), and the N^C^N-coordinated complex [Pt(bdq)(Cl)] (Hbdq = benzo[1,2-h:5,4-h’]diquinoline [55]. The rigid ligand backbones in these complexes, compared with the more flexible parent 6-phenyl-2,2′-bipyridyl (C^N^N) or dipyridyl-benzene (N^C^N) derivatives, leads to red-shifted photoluminescence with longer lifetimes and higher *Φ*_L_.

In a number of previous studies, the coligand (ancillary ligand) L in Pt(II) complexes with tridentate C^N^N, N^C^N, or C^N^C chromophores showed a marked impact on the luminescence properties [17,28,45,46,47,49,50,51,52,53,54,55,56]. This represents one of the benefits of the tridenate coordination approach for luminescent cyclometalated Pt(II) complexes, in comparison with the use of one tetradentate [17,22,23,24,25,42,43,52,53] or two bidentate ligands [5,11,39,51,57].

**Scheme 2:**
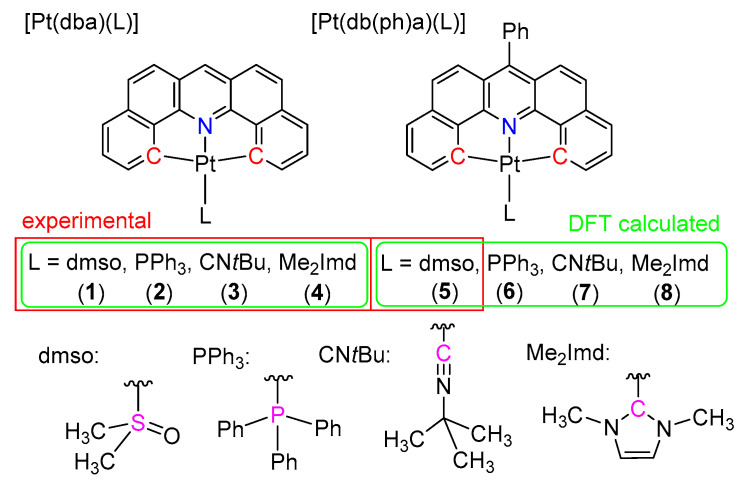
Schematic representation of the complexes [Pt(dba)(L)] (**1** to **4**) and [Pt(db(ph)a)(L)] (**5** to **8**).

Beginning with the previously reported complex [Pt(dba)(dmso)] (**1**) [33], we sought to improve the photoluminescence properties by exchanging the dmso coligand for PPh_3_, CN*t*Bu (*tert*-butyl-isocyanide), or Me_2_Imd (*N,N*’-dimethylimidazolydene) (complexes **2**, **3**, and **4**; Scheme 2). All three of the new coligands are expected to produce a stronger ligand field splitting (or ligand field stabilization energy) due to their superior σ-donating and π-accepting capabilities. This should destabilize the dark d-d* excited states further, and thus lead to superior luminescence quantum yields for the corresponding complexes. In addition to UV-vis absorption and time-resolved photoluminescence spectroscopy, we also studied the electrochemistry of the complexes, using cyclic voltammetry and spectroelectrochemical UV-vis spectroscopy, as well as modelling the excited states and electronic transitions employing (TD)-DFT calculations. The 7-phenyl substituted derivatives [Pt(db(ph)a)(L)] (H_2_db(ph)a = 7-phenyldibenzo[c,h]acridine) were also included in this study for comparison, as the experimental data for L = dmso (complex **5**) was already available [33], while further derivatives with L = PPh_3_, CN*t*Bu, and Me_2_Imd (**6**, **7**, and **8**) were calculated to probe if the 7-phenyl derivative of dba is potentially more interesting for phosphorescent Pt(II) complexes.

## 2. Results and Discussion

### 2.1. Synthesis, Analytical Characterization and Molecular Structures

**Scheme 3:**
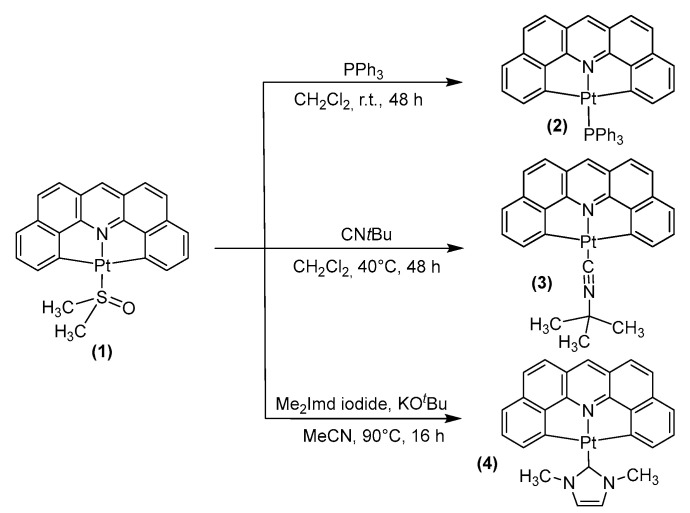
Synthesis of the complexes [Pt(dba)(L)] **2** to **4** from **1**.

The two-step synthesis of the complex [Pt(dba)(dmso)] from the H_2_dba protoligand and K_2_PtCl_4_ has previously been described [33]. The [Pt(dba)(dmso)] (**1**) was reacted with PPh_3_, CN*t*Bu and Me_2_Imd (*N,N*’-dimethylimidazolydene) to yield the three new complexes [Pt(dba)(L)] in excellent yields (93 to 98%) (Scheme 3, further details are found in the Section 3).

**Figure 1:**
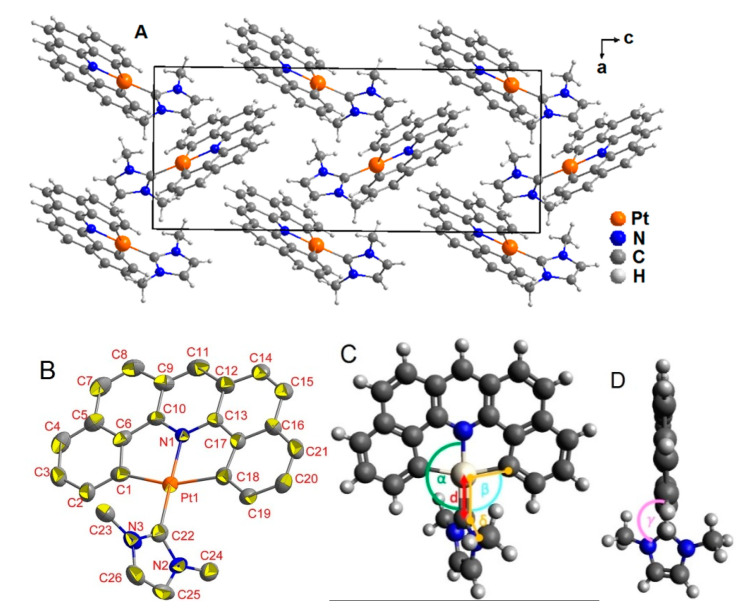
Crystal structure of [Pt(dba)(Me_2_Imd)] (**4**) viewed along the crystallographic *a* axis (**A**) and the molecular structure in the crystal (**B**) with thermal displacement ellipsoids at 50% probability and including the numbering; H atoms were omitted for clarity. DFT-optimized *S*_0_ structure of **4** (**C**,**D**) displaying the specific angles listed in Table 1.

All four compounds gave single crystals suitable for XRD structure analysis, and the crystallographic data is shown in Table S1 of the Supplementary Material (SM). All of the structures, with the exception of [Pt(dba)(Me_2_Imd)] (**4**), are characterized by pronounced head-to-tail π-stacks of two molecules each (Figure S1 to Figure S3, SM), which is typical for square planar complexes with planar tridentate or tetradentate ligands [19,21,25,26,27,28,29,33,34,35,36,39]. For **4**, the carbene ligand interrupts this interaction and a complex packing motive is observed (Figure 1 and Figure S4).

A closer inspection of the π-stacks showed that the centroid^...^centroid distance of the central pyridyl ring of [Pt(dba)(dmso)] constitutes approximately 3.62 Å, with an offset of approximately 1.25 Å, which lies in the range of typical π-interactions [45,55,56,58,59]. In contrast, for the PPh_3_ and the CN*t*Bu derivatives **2** and **3**, the bulky coligands enlarge these centroid^...^centroid distances, to 4.53 Å (0.93 Å offset) for **2** and to 5.59 Å (1.12 Å offset) for **3**. Therefore, dimerization through stacking in solution might have an impact on the photophysical properties only for the dmso complex (**1**). On the other hand, our previous study did not give evidence for this phenomenon [33].

The molecular structures show the expected square planar coordination of the d^8^ configured Pt(II) (Figure 1). The dba ligand is also completely planar, and the S, P, or C donor atoms of the ancillary ligands complete the planar coordination with N–Pt–X angles (α angle, Figure 1) of around 180°, while the residual atoms of the coligands fill the space at this fourth coordination site. The planar Me_2_Imd ligand in **4** shows a tilt angle towards the Pt,C,C,N coordination plane of about 58° (δ angle). The Pt–C–N unit (γ angle) in the CN*t*Bu complex **3** is almost linear (Table 1).

The optimized geometries of the singlet ground state *S*_0_ (Figure 1 and Figure S5) and the lowest excited triplet state *T*_1_ (Figure S6) were calculated by DFT methods. The *S*_0_ geometries are in good agreement with the experimental data from the X-ray diffractometry (Table 1). The *S*_0_ and *T*_1_ geometries hardly differ between all of the complexes; the largest differences occurred for the dmso and PPh_3_ derivatives, where the N–Pt–X and C–Pt–X angles change by a few degrees upon relaxation in the *T*_1_ state. In the case of PPh_3_, the planarity of the complex is broken in the *T*_1_ state, with the Pt(II) center clearly protruding out of the coordination plane. The Pt1–C1–C6–C10 dihedral angle (Figure 1) measures 7°.

### 2.2. Electrochemistry and DFT-Calculated Orbitals

**Figure 2:**
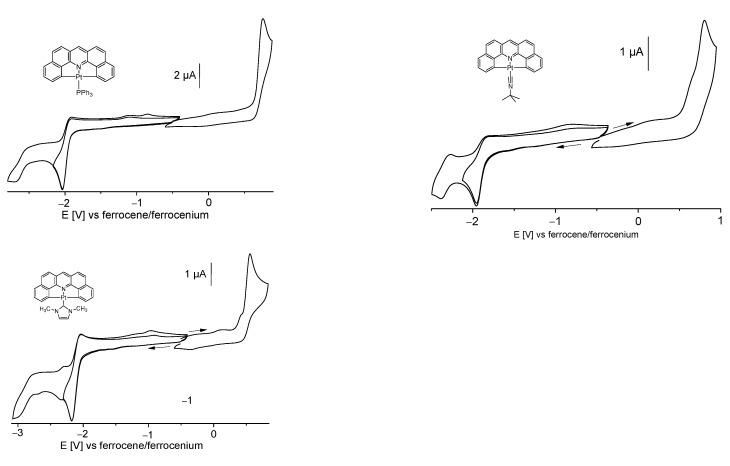
Cyclic voltammogramms of [Pt(dba)(L)] with L = PPh_3_ (**left**), CN*t*Bu (**middle**), and Me_2_Imd (**right**) in 0.1 M *n-*Bu_4_NPF_6_/THF.

On the first view, the complexes show irreversible oxidation processes ranging between around 0.55 and 1 V (Figure 2 and Figure S7, data in Table 2); based on the DFT calculations, they can be assigned to an essentially Pt-centered Pt(II)/Pt(III) couple. The DFT-calculated highest occupied molecular orbitals (HOMO) are energetically ordered in growing order as Me_2_Imd > PPh_3_ > CN*t*Bu > dmso (Figure S8). The electrochemical potentials increase along the same series for the dba complexes, which roughly match the expected increasing σ-donating strength of the coligand.

A first reduction is observed for all of the complexes at around −2 V. This process is partly reversible for the complexes **2** to **4**, but irreversible for the dmso complex **1** (Figure S7). Regardless of the slightly different reversibility, the very similar potentials point to a largely ligand π*(dba) centered reduction processes. This is in line with the DFT-calculated lowest unoccupied molecular orbitals (LUMO), which show the growing energetic sequence Me_2_Imd > PPh_3_ > CN*t*Bu > dmso (Figure S8); this correlates with the expected higher π-backbonding ability of the newly introduced coligands compared to dmso.

The irreversibility of both the oxidation and reduction processes were explained by rapid chemical reactions (*C*) following the electrochemical processes (*E*). If the two steps are timely correlated, this is called an *EC* mechanism or *EC* process [31,33,36,60,61]. Thus, secondary yet small, reduction waves found at −2.8 to −3.0 V represent the *EC* products. The introduction of the pending phenyl group led to markedly higher reduction and oxidation potentials for [Pt(db(ph)a)(dmso)], which is in line with the contribution of the dba core to the HOMO.

The electrochemical gaps increase along the series of dba complexes Me_2_Imd < CN*t*Bu < PPh_3_ < dmso following the trend of decreasing ligand strength (Table 2).

### 2.3. UV-Vis Absorption Spectroscopy

**Figure 3:**
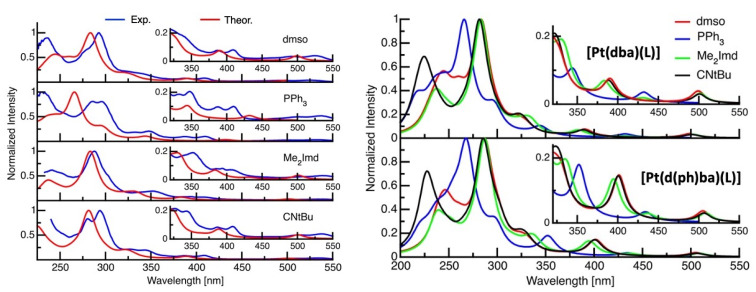
**Left:** TD-DFT-calculated UV-vis absorption spectra (red lines, Theor.) of [Pt(dba)(L)] for L = dmso, PPh_3_, Me_2_Imd, and CN*t*Bu in CH_2_Cl_2_ compared with the experimental spectra (black lines, Exp.). **Right:** Comparison of calculated UV-vis absorption spectra of [Pt(dba)(L)] and [Pt(d(ph)ba)(L)] for L = dmso, PPh_3_, Me_2_Imd, and CN*t*Bu.

The experimental UV-vis absorption spectra of the four [Pt(dba)(L)] complexes are very similar (Figure 3 and Figure S9). Four series of absorption maxima are discernible with very intense bands between 250 and 300 nm, along with two vibrationally structured medium-intense groups in the range between 300 and 420 nm, as well as a fourth structured band system between 450 and 550 nm that tails down to cut-offs (zero absorption) between 570 and 580 nm (Table 3).

The long-wavelength absorption and cut-off energies are only slightly smaller (by 0.03 to 0.05 eV) for the complexes with the new coligands, **2**, **3**, and **4**, compared with the parent dmso complex **1** (Table 3) and vary only slightly for these three complexes, in contrast to the marked differences in the electrochemical potentials and gaps (Table 2). The corresponding intensities are very different and the CN*t*Bu complex reaches not even half the intensities of the PPh_3_ derivative. Nevertheless, the similar band shapes and energies point to comparable excited states; we assume transitions into π–π* configurations for the two high-energy band systems (200 to 350 nm) and transitions into mixed π–π*(LC)/metal(d)-to-ligand(π*)(MLCT) states for the two long-wavelength absorption processes (350 to 550 nm), in line with previous reports [10,11,16,17,18,19,20,21,45,46,47,49,50,51,52,53,54,55,56,62]. We infer a relatively small metal participation from the rather low impact of the coligand exchange.

The recently reported complexes [Pt(naphen)(X)] (Hnaphen = naphtho[1,2-b][1,10]phenanthroline, X = Cl or C≡CPh) containing the rigid tridentate C^N^N-coordinating pericyclic naphen ligand and the derivatives [Pt(thnaphen)(X)], bearing the slightly more flexible thnaphen^−^ unit (Hthnaphen = 5,6,8,9-tetrahydro-naphtho[1,2-b][1,10]phenanthroline), show long-wavelength absorption bands between 523 and 534 nm [55], and thus appear comparable to the dba complexes. In contrast to this, the N^C^N-coordinated complex [Pt(bdq)(Cl)] containing the rigid bdq^−^ ligand (Hbdq = benzo[1,2-h:5,4-h’]diquinoline) absorbs markedly blue-shifted (493 nm), which is reasonable for the comparison between the C^N^N and the N^C^N coordination pattern [16,36,55,63].

### 2.4. TD-DFT Calculated Absorption Spectra

The most intense calculated transitions are found in the range between 225 and 325 nm (Figure 3, left). The maxima for the complexes with L = dmso and Me_2_Imd show very good agreement with the experimental data, while deviating by only 4 to 9 nm, whereas for PPh_3_ and CN*t*Bu, the deviation is somewhat more pronounced (ca. 25 nm).

The main peak of the [Pt(dba)(dmso)] complex has a maximum of approximately 283 nm and corresponds to a transition into a state with dominant contributions from HOMO−1→LUMO+1 (32%), HOMO−3→LUMO+3 (10%) and HOMO−7→LUMO excitations (10%). The [Pt(dba)(PPh_3_)] complex has its highest peak at 266 nm, leading to an excited state with contributions from HOMO−1→LUMO+1 (48%), HOMO−5→LUMO (13%), and HOMO−7→LUMO (13%) excitations. For [Pt(dba)(Me_2_Imd)], this peak is located at 277 nm and is associated with a state described with contributions of HOMO−2→LUMO+2 (23%), HOMO−7→LUMO (23%), HOMO−3→LUMO+2 (14%), and HOMO-6→LUMO (12%) excitations. For [Pt(dba)(CN*t*Bu)], the maximum can be found at 282 nm, and it is dominated by a transition into an excited state with contributions from HOMO−1→LUMO+2 (51%), HOMO→LUMO+3 (25%) and HOMO−7→LUMO (14%) excitations.

When comparing the TD-DFT-calculated UV-vis absorption spectra of the complexes [Pt(dba)(L)] and [Pt(db(ph)a)(L)] (5 to 8), we found a red-shift of the main maximum when going from PPh_3_ (**6**) via CN*t*Bu (**7**) to Me_2_Imd (**8**) and dmso (**5**) and a coincidence of the main peaks for the Me_2_Imd and DMSO complexes in both cases. In addition, for the long-wavelength absorption bands, there is only a very small difference between the spectra of the [Pt(dba)(L)] and [Pt(db(ph)a)(L)] complexes (Figure 3, right).

### 2.5. Time-Resolved Photoluminescence Spectroscopy

At room temperature and in diluted CH_2_Cl_2_ solutions, the four dba Pt(II) complexes **1** to **4** show a red luminescence with a broad band centered at around 600 nm (Figure S10), most likely arising from the mixed ^3^LC/^3^MLCT excited states; this is in line with previous reports [10,11,16,17,18,19,20,21,45,46,47,49,50,51,52,53,54,55,56,62], as well as with our TD-DFT calculations (*vide infra*). The change of the monodentate coligand does not substantially influence the emission profile of the complexes; only the vibrational progression is marginally affected. The absence of ^3^O_2_ in the Ar-purged solutions generally increased the lifetime of the complexes, compared to the air-equilibrated solutions, in line with a triplet character of the luminescence (Table 4). The most remarkable finding was that the replacement of L = dmso by the stronger ligands PPh_3_, CN*t*Bu, and Me_2_Imd had almost no effect on the emission energies and a rather detrimental effect on *Φ*_L_, which decreases along the series of coligands dmso >> CN*t*Bu >> Me_2_Imd > PPh_3_ and is generally lower for the complexes with stronger coligands L than for the parent complex [Pt(dba)(dmso)]. In the more concentrated solutions, we observed red-shifted emissions which were likely a result of the aggregation of the molecules. However, in this report we wanted to focus on the luminescence properties of the isolated molecules and avoided aggregation by using low concentrations (*c* = 10^−5^ M). In future studies, we will investigate the aggregation of these complexes in solution and in solid materials.

The short-lived component of the adjusted biexponential decays for *τ* in air-equilibrated CH_2_Cl_2_ solutions are due to traces of the free H_2_dba ligand (*τ*_dba_ ≈ 5 ns) (Table 4). However, in most cases, this effect was not seen in Ar-purged solution due to the vast increment of *Φ*_L_ for the complexes under these conditions. The fluorescence quantum yield of dba is not negligible in CH_2_Cl_2_ at 298 K (*Φ*_L_ = 0.44); thus, even traces of the ligand are detected in case of the poorly phosphorescent complexes in the presence of oxygen. Therefore, a trace amount of the ligand below 0.01% might escape the analysis by NMR, elemental analysis and other methods, while still being detectable through intrinsic fluorescence. Importantly, the studied dba Pt(II) complexes were purified by column chromatography, and NMR spectra show no signals for the H_2_dba precursor (with the exception of the PPh_3_ complex, see Figures S31–S35 in the Supporting Material). We assume that, in addition to the complexes as molecular species, small stacks (see crystal structures) of complexes with the ligand precursor H_2_dba are also eluted. Our findings also emphasize that special care has to be taken when measuring weakly phosphorescent complexes stemming from a brightly fluorescent ligand precursor, in order to avoid potential confusion with dual emission [55].

**Figure 4:**
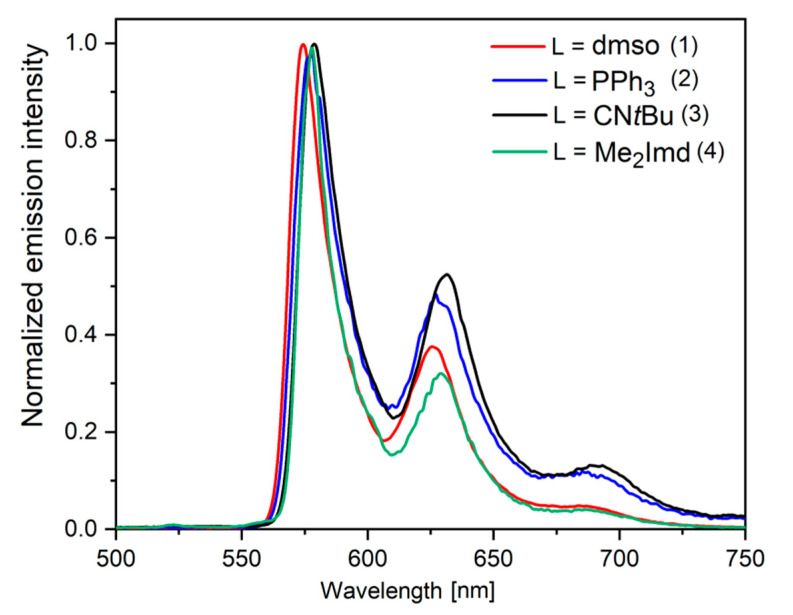
Normalized photoluminescence spectra of the [Pt(dba)(L)] complexes (**1** to **4**) in a frozen CH_2_Cl_2_:MeOH 1:1 glassy matrix at 77 K (λ_ex_ = 350 nm, *c* = 10^−5^ M). The corresponding excitation spectra as well as the emission of the dba ligand can be found in the SM, Figures S11–S15.

In frozen glassy matrices at 77 K, the charge-transfer states are destabilized, which lowers the MLCT character of the emissive *T*_1_ state, while causing a blue-shifted photoluminescence (576 nm for the dmso complex). Therefore, the observed emission at 77 K (Figure 4) predominantly originates from ligand-centered triplet states (^3^LC), as reflected by the enhanced vibrational progression, compared with 298 K (Figure S10). Under these conditions, all of the complexes show prolonged excited state lifetimes and quantum yields between 0.8 and 1.0 due to a diminished *k*_nr_ (Table 4). In addition, it is worth noting that at 77 K, diverse comparably stable conformations can be locked in the glassy matrix, leading to biexponential decays, as was previously observed for other Pt(II) complexes [22,31,32,55,56,62].

In addition, *Φ*_L_ and *τ* at 77 K were used to determine the radiative (*k*_r_) and radiationless (*k*_nr_) deactivation rate constants (Table 4), according to equations 1 to 4, and assuming a unitary intersystem crossing efficiency for *S*_1_→*T*_n_→*T*_1_, the following relations are valid

(1) $$k_{r}=\frac{\Phi_{L}}{\tau }$$

(2) $$k_{\mathrm{nr}}=k_{{\mathrm{ISC}}^{\prime }}$$

(3) $$\tau =\frac{1}{k_{r}+k_{\mathrm{nr}}\;}$$

(4) $$k_{\mathrm{nr}}=\frac{1-\Phi_{L}}{\tau \;}$$

where *k*_IC_ is the internal conversion rate constant and *k*_ISC′_ is the intersystem crossing rate constant (*T*_1_→*S*_0_). For biexponential decays, amplitude-weighted average lifetimes (*τ*_av_amp_) were used to estimate the rate constants [64].

Depending on the nature of the coligand, minor changes in both *k*_r_ and *k*_nr_ are found (Table 4). In a frozen glassy matrix, at 77 K, the complexes are trapped in their most stable conformation(s) within the solid and the differences in solvation environments and rotovibrational relaxation effects are diminished; therefore, only minor differences in *k*_r_ and *k*_nr_ can be found.

For the complexes [Pt(naphen)(X)] (X = Cl or C≡CPh) with the rigid C^N^N-coordinating naphen^−^ ligand, or the slightly more flexible tetrahydro-derivative [Pt(thnaphen)(X)], similar broad PL spectra were found at 298 K in the CH_2_Cl_2_ solution, peaking at 630 nm for the naphen and 570 nm for the thnaphen derivatives [55], i.e., slightly red-shifted with respect to the dba complexes. The N^C^N-coordinated complex [Pt(bdq)(Cl)] containing the rigid bdq^−^ ligand shows a slightly more pronounced vibronic structure and a maximum at 568 nm [55]. The quantum yields *Φ*_L_ ranged between 0.08 and 0.32, and are similar to the dba complexes.

At low *T* (77 K, frozen glassy matrices), vibrationally structured emission profiles were found for these complexes, and they appear similar to those observed for the dba complexes, but with slightly higher emission energies for the naphen (600 nm) and thnaphen (530 nm) complexes, yet with almost no shift for the bdq complex. The *Φ*_L_ values were close to 1 for these complexes [55]. Taking into account the photoluminescence lifetime of the [Pt(naphen)(Cl)] complex, we assign the phosphorescence to mixed ^3^LC/^3^MLCT excited states with only a small metal contribution and dominant ^3^LC character [55].

### 2.6. DFT-Calculated Emission Spectra

**Figure 5:**
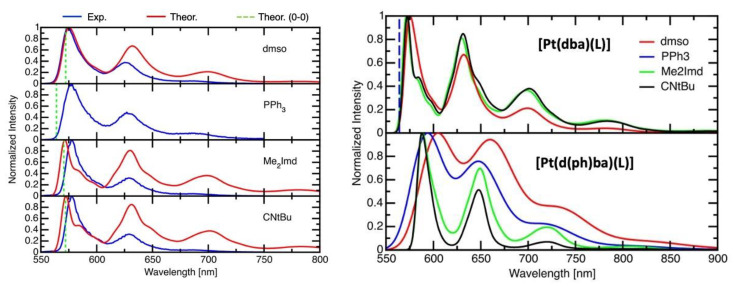
**Left:** DFT-calculated emission spectra (red lines, Theor.) of [Pt(dba)(L)] for L = dmso, PPh_3_, Me_2_Imd, and CN*t*Bu in CH_2_Cl_2_ compared with the respective experimental data at 77 K (blue lines, Exp.). The calculated 0-0 transitions are shown as vertical green dashed lines. **Right:** Comparison of calculated emission spectra of [Pt(dba)(L)] and [Pt(d(ph)ba)(L)].

The calculated vibrationally resolved emission spectra (red lines) and the zero-point corrected 0-0 emission energies (green dashed lines) are in very good agreement with the experimental data (blue lines). For all of the complexes (Figure 5, left), the main peak and the most intense shoulder deviate by less than 10 nm (except for PPh_3_). Nonetheless, for the calculated 0-0 energy of the PPh_3_, the agreement is still reasonable. A vibrationally resolved emission spectrum could not be computed for PPh_3_ due to numerical issues (see Computational Details).

When comparing the complexes [Pt(dba)(L)] and [Pt(db(ph)a)(L)] for the different coligands (Figure 5, right), the main peak shifts to shorter wavelengths (for both cases) when the dmso ligand is replaced by Me_2_Imd. In the case of [Pt(db(ph)a)(L)], the main emission peak for PPh_3_ lies inbetween the other two. Interestingly, the vibronic coupling pattern is very different for the two C^N^C chelate ligands and the change from dba to db(ph)a causes a general, yet slight, red-shift.

**Figure 6:**
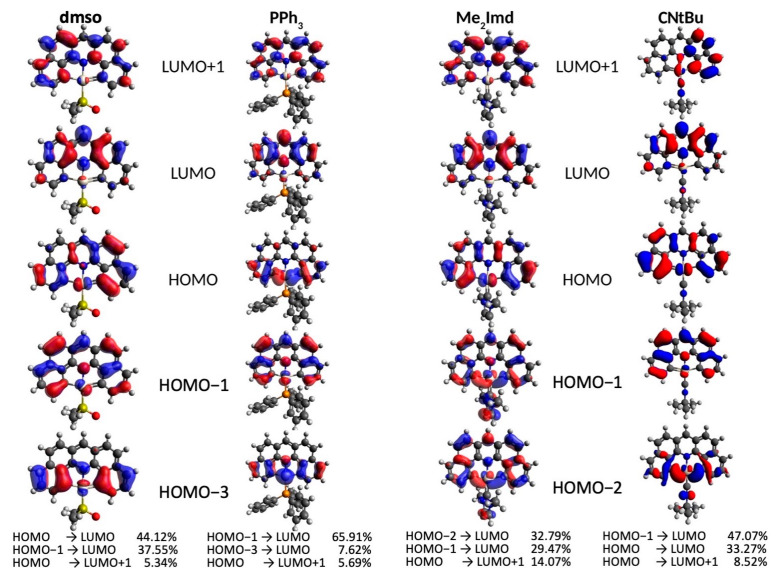
Frontier orbitals of complex [Pt(dba)(L)] with L = dmso, PPh_3_, Me_2_Imd, CN*t*Bu at the optimized *T*_1_ geometry together with the highest contributions of monoelectronic excitations to the *T*_1_ state (in %).

The calculated orbitals contributing to the description of the emissive *T*_1_ state show that the HOMO and LUMO are partially localized on the metal ion, as well as on the ligand scaffold (Figure 6). For Me_2_Imd, partial contributions from the coligand can also be identified. In general, the occupied orbitals have higher metal contributions than their unoccupied counterparts. For the dmso complex, exclusively, the *T*_1_ state can be described as a predominant HOMO→LUMO excitation, which involves a significant amount of charge transfer between the metal and the ligand (Figure 6).

However, for all of the complexes, the *T*_1_ state is composed of a number of monoelectronic excitations. For the dmso complex, there are two main contributions to the *T*_1_ state, namely, HOMO→LUMO (44.1%) and HOMO−1→LUMO (37.6%). In addition, there is a small contribution from the HOMO→LUMO+1 excitation (5.3%). The PPh_3_ derivative shows a major contribution (65.9%) from the HOMO−1→LUMO excitation. Other smaller contributions come from HOMO−3→LUMO (7.6%) and HOMO→LUMO+1 (5.7%). The Me_2_Imd complex exhibits two approximately equal contributions from HOMO−2→LUMO (32.8%) and HOMO−1→LUMO (29.5%), as well as a significant contribution from the HOMO→LUMO+1 (14.1%) excitation. In the case of CN*t*Bu, the main contributions stem from the HOMO−1→LUMO (47.1%), HOMO→LUMO (33.3%), and HOMO→LUMO+1 (8.5%) excitations. Generally speaking, for all of the complexes, the character of the *T*_1_ state can be described as a mixture of intraligand LC and MLCT character; this is in line with our initial assumptions.

**Figure 7:**
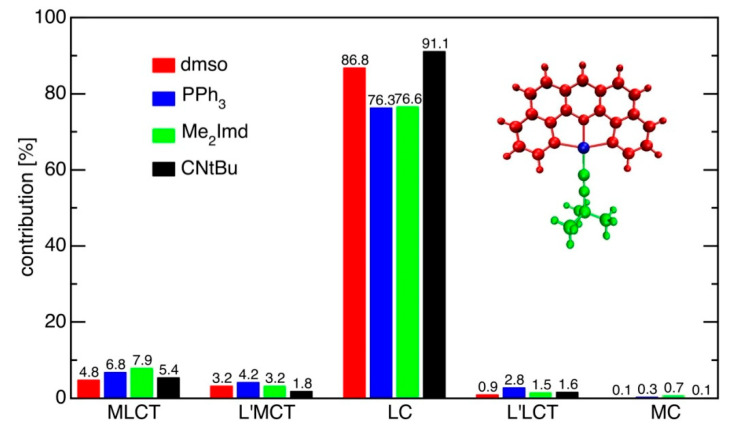
Decomposition of the *T*_1_ state for [Pt(dba)(L)] with L = dmso (red), PPh_3_ (green), Me_2_Imd (black), CN*t*Bu (blue) into contributions originating from MLCT, L’MCT, LC, L’LCT and MC configurations determined from a correlated electron-hole pair analysis [48]. Right: Partitioning used for the example of [Pt(dba)(CN*t*Bu)].

A quantitative description of the *T*_1_ character was obtained through a correlated electron-hole pair analysis [65]. The resulting decomposition into LC (π−π*), MLCT (d−π*), coligand-to-dba charge transfer (L’LCT), coligand-to-metal charge transfer (L’MCT), and MC (d−d*) contributions (Figure 7) is based on a partitioning of the complexes into the dba ligand, the metal and the coligand (as shown for **3** in Figure 7).

Overall, the analysis shows that all of the complexes exhibit a similar character for the *T*_1_ state. The dmso, PPh_3_, Me_2_Imd, and CN*t*Bu complexes all predominantly show a LC character (86.8%, 76.3%, 76.6%, and 91.1%, respectively), and only a small degree of CT character. The proportion of the MLCT character increases from dmso (4.8%) via CN*t*Bu (5.4%) and PPh_3_ (6.8%) to Me_2_Imd (7.9%).

The high LC character of the excited states explains our spectroscopic findings and helps us to understand the marginal influence of the coligands L on the PL properties. However, it cannot explain why the change from dmso to the stronger σ-donating and π-accepting ligands PPh_3_, CN*t*Bu and Me_2_Imd drastically reduces the *Φ*_L_ at 298 K, namely, from a remarkable 0.23 (dmso) to almost zero for PPh_3_. At the same time, at 77 K, the introduction of the new ligands led to increased *Φ*_L_ values, with the highest performance observed for the PPh_3_ complex. In future work, we will seek insights into the dynamics of the excited states by dedicated calculations. In fact, the results depicted in Figure 7 only show the composition concerning the initial excited states [65], which might change over time, as recent work on related luminescent complexes has revealed [66,67].

### 2.7. Spectroelectrochemistry (SEC)

**Figure 8:**
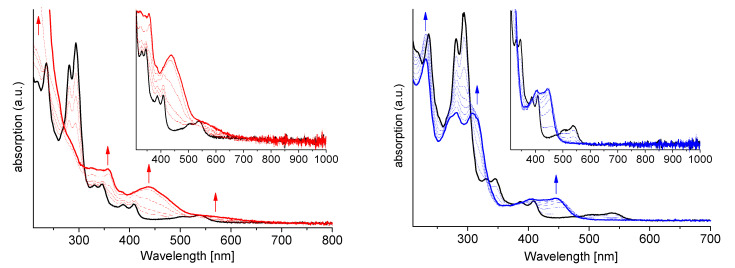
UV-vis absorption spectra of [Pt(dba)(CN*t*Bu)] (**3**) in THF/*n*-Bu_4_NPF_6_, recorded during cathodic reduction (left, traces of reduced species in red) and during anodic oxidation (right, traces of oxidized species in blue)-spectroelectrochemistry.

The UV-vis spectroscopic response upon cathodic reduction and anodic oxidation (SEC) was studied for all of the for complexes in the THF/*n-*Bu_4_NPF_6_ solution (Figure 8, more figures and data in the SM).

First of all, the SEC experiments show that the initial reductions and oxidations of all four compounds occur, irreversibly, on the timescale of minutes. In the CV experiments, the first reduction showed some degree of reversibility, but the CV experiment is on a timescale of seconds. Nevertheless, the spectral responses of the compounds upon reduction or oxidation leave the impression that defined *EC* (electrochemical + subsequent chemical reaction) [61,68,69,70] reactions occur. This assumption is derived from the almost isosbestic character of the produced spectral traces (Figure 8).

Upon reduction, a pronounced band, at around 435 nm, was observed for the set of reduced species, while long-wavelength bands at around 650 nm were only observed for the dmso (**1**) and Me_2_Imd (**4**) complexes. Assuming a ligand π*-centered reduction, we were surprised to see no long-wavelength transitions into π*−π* states, which are usually observed for complexes with reduced heteroaromatic ligands [27,36,68,69,70,71,72]. They most likely occur for the dba system at wavelengths below 1100 nm, beyond the detection limit of our spectrometer.

The oxidation of the four complexes leaves UV-vis absorption traces that are very dissimilar, thus confirming the mixed metal/coligand character of the oxidation process. Importantly, no evidence for stable or transient Pt(III) species was observed, in line with a rapid chemical reaction after oxidation (*EC*). Bands for Pt(III) species are expected in the range 500 to 1800 nm [72].

## 3. Experimental Section

### 3.1. General Information

Commercially available chemicals were purchased from Sigma-Aldrich, Acros, ABCR or Fisher-Scientific and were used without further purification. Dry THF was obtained from distillation over sodium/potassium alloy. All reactions and measurements involving metal complexes were conducted under argon, using standard Schlenk techniques. The complex [Pt(dba)(dmso)] (**1**) was synthesized and characterized as previously described [33]. Finally, 1,3-dimethylimidazolium iodide was prepared from commercially purchased methylimidazole and methyl iodide as described in the literature [73].

### 3.2. Syntheses

#### 3.2.1. Synthesis of [Pt(dba)(PPh_3_)] (**2**)

A mixture of 40.14 mg [Pt(dba)(dmso)] (0.073 mmol, 1 eq.) and PPh_3_ (20.65 mg, 0.079 mmol, 1.1 eq.) was dissolved in 30 mL CH_2_Cl_2_ and the reaction mixture was stirred for 96 h at ambient temperature. The solvent was evaporated under reduced pressure and a red solid was obtained. The purification was conducted by column chromatography on silica (CH_2_Cl_2_/*n*-hexane 1:1). The product was obtained as a red solid (56.25 mg, 0.071 mmol, 97%). Elemental analysis found (calculated for C_39_H_26_NPPt, M_W_ = 734.68 g mol^−1^): C, 63.78 (63.76); H, 3.55 (3.57); N, 1.92 (1.91). ^1^H NMR (600 MHz, CD_2_Cl_2_): δ (ppm) = 8.50 (s, 1H), 7.96 (t, *J* = 6.3 Hz, 6H), 7.63 (d, *J* = 6.3 Hz, 2H), 7.58 (d, *J* = 8.9 Hz, 2H), 7.50 (t, *J* = 7.3 Hz, 3H), 7.44 (t, *J* = 7.5 Hz, 6H), 7.38 (d, *J* = 7.7 Hz, 2H), 7.04 (t, *J* = 7.5 Hz, 2H), 6.29 (d, 2H). ^31^P NMR (234 MHz, CD_2_Cl_2_): δ (ppm) = 25.66 (s, ^1^*J*_Pt-P_ = 8209 Hz). HR-ESI-MS(+) (MeOH) calculated for [M]^+^ *m*/*z* = 734.1454, found 734.15015; calculated for [dba+H]^+^ *m*/*z* = 280.112624, found: 280.113714. UV-vis absorption (CH_2_Cl_2_): λ (nm) ε (10^3^ M^−1^cm^−1^) = 296 (50.2), 347 (12.7), 409 (6.2), 532 (2.1).

#### 3.2.2. Synthesis of [Pt(dba)(CN*t*Bu)] (**3**)

A mixture of 40.14 mg [Pt(dba)(dmso)] (0.073 mmol, 1 eq.) and CN*t*Bu (8.3 μL, 0.073 mmol, 1 eq.) was dissolved in 20 mL CH_2_Cl_2_ and stirred for 24 h at 50 °C. The solvent was evaporated under reduced pressure. The product was obtained as a red solid (39.7 mg, 0.072 mmol, 98%). Elemental analysis found (calculated for C_26_H_20_N_2_Pt, M_W_ = 555.53 g mol^−1^): C, 56.22 (56.21); H, 3.65 (3.63); N, 5.08 (5.04). ^1^H NMR (600 MHz, CD_2_Cl_2_): δ (ppm) = 8.36 (s, 1H), 7.68 (dd, *J* = 2.6 Hz, 2H), 7.57 (d, *J* = 8.9 Hz, 2H), 7.47 (d, *J* = 8.9 Hz, 2H), 7.45–7.41 (m, 4H), 1.67 (s, 9H). HR-ESI-MS(+) (MeOH) calculated for [M]^+^ *m*/*z* = 555.127440, found 555.13287. UV-vis absorption (CH_2_Cl_2_): λ (nm) ε (10^3^ M^−1^cm^−1^) = 294 (27.0), 343 (5.2), 408 (2.4), 533 (1.0).

#### 3.2.3. Synthesis of [Pt(dba)(Me_2_Imd)] (**4**)

A solution of 40.14 mg [Pt(dba)(dmso)] (0.073 mmol, 1 eq.) was dissolved in 20 mL MeCN. Freshly synthesized 1,3-dimethylimidazolium iodide (17.2 mg, 0.077 mmol, 1.05 eq.) and KO*t*Bu (9.83 mg, 0.088 mmol, 1.2 eq.) were added. The solution was heated under reflux at 90 °C for 16 h. After the solvent was reduced to the half volume, the product was precipitated by addition of 20 mL water. After filtration, the crude solid was washed with toluene to obtain the product as a red solid (38.61 mg, 0.068 mmol, 93%). Elemental analysis found (calculated for C_26_H_19_N_3_Pt, M_W_ = 568.53 g mol^−1^): C, 54.95 (54.93); H, 3.38 (3.37); N, 7.39 (7.39). ^1^H NMR (600 MHz, CD_2_Cl_2_): δ (ppm) = 8.35 (s, 1H), 7.55 (d, *J* = 8.9 Hz, 2H), 7.49 (d, *J* = 8.9 Hz, 2H), 7.40 (d, *J* = 7.8 Hz, 2H), 7.30 (t, *J* = 7.3 Hz, 2H), 7.23 (d, *J* = 6.8 Hz, 2H), 7.01 (t, *J* = 5.4 Hz, 2H), 5.78 (s, 6H). EI-MS(+) (MeOH) calculated for [M]^+^ *m/z* = 568.122689, found: 568; calculated for [Ptdba]^+^ *m*/*z* = 472.053940, found 472; calculated for [dba]^+^ 279.104799, found 279. UV-vis absorption (CH_2_Cl_2_): λ (nm) ε (10^3^ M^−1^cm^−1^) = 300 (24.8), 352 (8.9), 406 (3.2), 536 (1.3).

### 3.3. Instrumentation

^1^H and ^31^P **NMR spectra** were recorded on a Bruker Avance II 600 MHz spectrometer (^1^H: 600.13 MHz, ^31^P: 242.88 MHz) with a triple resonance (TBI) 5 mm inverse probehead with z-gradient coil using a triple resonance. Chemical shifts are relative to TMS, respectively. **UV-vis absorption spectra** were recorded with Varian Cary 05E or Cary 50 scan spectrophotometers. **Photoluminescence spectra** at room temperature and at 77 K were recorded with a PicoQuant FluoTime 300. The same equipment was used for lifetime measurements. **Lifetime analysis** was performed using the commercial EasyTau 2.2 software. The quality of the fit was assessed by minimising the reduced chi-squared function. **Photoluminescence quantum yields** were determined with a Hamamatsu Photonics absolute PL quantum yield measurement system (C9920–02), equipped with a L9799-01 CW Xenon light source, monochromator, photonic multichannel analyser and integrating sphere (error of maximum ± 2% for *Φ*_L_ is estimated). All of the solvents were of spectroscopic grade and were degassed prior to use. **Elemental analyses** were obtained using a HEKAtech CHNS EuroEA 3000 analyzer. **EI-MS** spectra were measured with a Finnigan MAT 95, and **HR-ESI-MS** using a THERMO Scientific LTQ Orbitrap XL. MS Simulations were performed using ISOPRO 3.0. **Electrochemical measurements** were carried out in 0.1 M *n-*Bu_4_NPF_6_/THF solution using a three-electrode configuration (glassy carbon working electrode, Pt counter electrode, Ag/AgCl reference electrode) and a Metrohm Autolab PGSTAT30 potentiostat and function generator. The ferrocene/ferrocenium couple served as internal reference.

### 3.4. X-ray Diffractometric Analysis of Single Crystals for Structure Determination

The crystals of **1** to **4** were obtained via isothermic evaporation at 0 °C from acetone/Et_2_O mixtures (v:v = 2:1). The measurement of [Pt(dba)(Me_2_Imd)] was performed using graphite-monochromated Mo-K_α_ radiation (*λ* = 0.71073 Å) with an IPDS 2T diffractometer (STOE and Cie.) at 100 K. [Pt(dba)(dmso)] and [Pt(dba)(CN*t*Bu)] were measured on a D8 Advance diffractometer (Bruker), with the same wavelength, at 100 K. Diffraction data of [Pt(dba)(PPh_3_)] were collected at the beamline P24 of PETRA III at the German Electron Synchrotron facility DESY using a wavelength of *λ* = 0.56076 Å and at a temperature of 100 K. The data were recorded on a Pilatus 1M CdTe detector. All of the structures were solved by dual space methods (SHELXT-2015) [74] and refined by full-matrix least-squares techniques against *F*^2^ (SHELXL-2017/1) [75,76] The non-hydrogen atoms were refined with anisotropic displacement parameters, without any constraints. The hydrogen atoms were included by using appropriate riding models. The numerical absorption correction of the data of [Pt(dba)(Me_2_Imd)] (X-RED V1.31; STOE and Cie, 2005, Darmstadt, Germany) were performed after optimising the crystal shapes using the X-SHAPE V1.06 (STOE & Cie, 1999, Darmstadt, Germany) [77,78]. For the data set acquired with synchrotron radiation, no absorption correction was omitted. Residual electron density in the pores alongside the crystallographic *b*-axis of [Pt(dba)(PPh_3_)] was removed. The crystallographic details are summarized in Table S1. Data of the structure solutions and refinements can be obtained for [Pt(dba)(dmso)] (CCDC 2141888 (100 K) and 2141889 (298 K)), [Pt(dba)(PPh_3_)] (CCDC 2070867), [Pt(dba)(CN*t*Bu)] (CCDC 2070866), and [Pt(dba)(Me_2_Imd)] (CCDC 2070868) free of charge at https://www.ccdc.cam.ac.uk/structures/ or from the Cambridge Crystallographic Data Centre, 12 Union Road, Cambridge, CB2 1EZ UK (fax: +44-1223 336033 or e-mail: deposit@ccdc.cam.ac.uk).

### 3.5. Computational Details

#### 3.5.1. Vibrationally Resolved Emission Spectra

The quantum-chemical calculations of the vibrational Franck-Condon spectra and optimized geometries were carried out with the quantum chemistry package, Gaussian 09 Rev. D.01 [79]. The CAM-B3LYP functional [80] was used in the density functional theory (DFT) calculations and the SDD basis was set, which applies an effective core potential for the Pt atoms [81] and the D95 basis set for H, C, N and O atoms [82]. The vibrational Franck-Condon spectra were calculated according to the method of Barone et al. [83,84,85], with Kohn-Sham DFT-based geometry optimizations in the *S_0_* and *T_1_* states, followed by frequency analysis calculations.

The energies were corrected by zero-point vibrational energies and thermal free energy contributions. In order to calculate the overlap integrals for the vibronic spectra, the transitions are divided into classes *C_n_*, where *n* is the number of the excited normal modes in the final electronic state. The maximum number of quanta per mode was set to 100 and the maximum number of quanta for combinations of two modes to 65. The number of integrals calculated was limited to 1.5 × 10^8^. A maximum of 20 classes were computed. The line spectrum was broadened using Gaussian functions with a half-width at half-maximum of 500 cm^−1^. The solvent CH_2_Cl_2_ was taken into account by the polarizable continuum model (PCM) in an integral equation formalism framework [86] with atomic radii from the universal force field model (UFF) [87].

#### 3.5.2. Character of the *T*_1_ State

The character of the emissive *T*_1_ state is determined by TD-DFT calculations, including 20 excited singlet and triplet states at the *T*_1_ geometry, optimized with Kohn-Sham DFT with multiplicity 3.

#### 3.5.3. Absorption Spectra

To obtain the UV-vis absorption spectrum of the complexes, TD-DFT calculations of the 40 lowest excited singlet states were performed with the PBE0 functional [88] and the SDD basis set. A Lorentzian broadening with a half-width at a half-maximum (HWHM) of 10 nm was used for each transition.

## 4. Conclusions

Starting from the previously reported complex [Pt(dba)(dmso)], containing the rigid dianionic, bis-cyclometalating, tridentate ^–^C^N^C^–^ ligand dba^2–^ (H_2_dba = dibenzo[c,h]acridine), we synthesized further derivatives [Pt(dba)L] with L = PPh_3_, CN*t*Bu and Me_2_Imd (*N,N*’-dimethylimidazolydene) as coligands (ancillary ligands) to improve their luminescence properties through the introduction of these stronger σ-donating and π-accepting ligands as compared to dmso.

Cyclic voltammetry showed partially reversible reduction waves ranging between −1.89 and −2.10 V and irreversible oxidation processes between 0.55 (L = Me_2_Imd) and 1.00 V (dmso), depending on the ancillary ligand L. The strongly variable oxidation potentials raised the electrochemical gaps from 2.65 to 2.91 V along the series Me_2_Imd < CN*t*Bu < PPh_3_ < dmso along the trend of decreasing ligand strength.

All four dba complexes partially show vibrationally structured long-wavelength absorption bands peaking at around 530 nm. TD-DFT calculated absorption spectra are in good agreement with the experimental findings, showing that they are slightly red-shifted, but perfectly reproduce the observed trends. The stronger coligands only cause a minor red-shift of the long-wavelength absorption bands (0.03 to 0.05 eV). The photoluminescence spectra of all four compounds are very similar and strongly depend on the presence or absence of ^3^O_2_, in line with the triplet character of the emissive state. In a solution at 298, they show broad bands with a minor vibronic structure and maxima at around 590 nm; in frozen glassy matrices, slightly blue-shifted (~580 nm) bands with vibrational progression were found. In contrast to the comparable emission energies, the photoluminescence quantum yields *Φ*_L_ differ between the four complexes and the lifetimes increase along the series Me_2_Imd < CN*t*Bu < PPh_3_ < dmso from 0.06 to 14 µs; surprisingly, the dmso complex constitutes the most efficient emitter, at 298 K (*Φ*_L(Ar)_ = 0.24). This is highly counterintuitive, as the ligand strength is considered to decrease from Me_2_Imd to dmso, as supported by the electrochemical results. In frozen glassy matrices at 77 K, the lifetimes are comparable and increase from 17 µs for the Me_2_Imd complex, to approximately 30 µs for the other three derivatives. The TD-DFT calculated emission spectra agree well with the experimental data for all of the complexes. Decomposition of the excited *T*_1_ states showed mostly ligand-centered (LC) contributions (80 to 90%) to the description of the emissive states, which is in line with the vibrationally structured emission bands, a minor metal-to-ligand charge transfer (MLCT) character (5 to 8%), marginal coligand-to-metal (L’MCT) or coligand-to-chelate ligand charge transfer (L’LCT) contributions and virtually no metal-centered (MC) character.

The exclusively theoretical study on the dba-7-phenyl substituted derivatives [Pt(db(ph)a)(L)] (H_2_db(ph)a = 7-phenyldibenzo[c,h]acridine) with L = PPh_3_, CN*t*Bu, and Me_2_Imd showed that these complexes were not superior to the dba congeners, in line with the previous experimental comparison of [Pt(db(ph)a)(dmso)] with the dba derivative.

The far lower *Φ*_L_ for the complexes containing the stronger coligands Me_2_Imd, CN*t*Bu, and PPh_3_ (in comparison with the parent dmso complex [Pt(dba)(dmso)] in solution at 298 K) is an unexpected result as most of the previous studies showed that the exchange of the coligand by a stronger σ-donor and/or π-acceptor improved the photoluminescence (i.e., higher quantum yields). However, at 77 K in frozen glassy matrix, the “ligand effect” was found as expected (higher *Φ*_L_ for stronger ligands). The elucidation of the cause for these surprising observation at 298 K is the object of ongoing research. At the same time, the assessment of their suitability for optoelectronic devices will require in-depth photophysical studies in the solid state, e.g., in polymeric matrices, which will be carried out in the near future. This will also include a study of the aggregation of these complexes in solution and in solid materials.

## Figures and Tables

**Table 1:** Selected data of experimental and DFT-optimized geometries in the *S*_0_ and *T*_1_ states for [Pt(dba)(L)] ^a^.

	State	dmso(1)	PPh_3_(2)	CN*t*Bu(3)	Me_2_Imd(4)
*d* Pt-X [Å]	exp.	2.19(7)	2.23(2)	1.89(3)	1.99(1)
	calc. *S*_0_	2.34	2.35	1.92	1.99
	calc. *T*_1_	2.35	2.39	1.92	1.99
*α* = N-Pt-X [°]	exp.	173.2(6)	176.2(1)	177.6(1)	177.8(4)
	calc.*S*_0_	174.1	176.7	179.8	179.7
	calc. *T*_1_	179.1	177.4	179.8	179.8
*β* = C-Pt-X [°]	exp.	94.47(7)	96.8(1)	102.9(1)	101.9(4)
	calc. *S*_0_	94.7	97.1	100.4	100.3
	calc. *T*_1_	99.4	99.2	100.4	100.4
*γ* = Pt-X-Y [°]	exp.	115.7(9)	113.4(2)	177.4(3)	128.7(9)
	calc. *S*_0_	115.1	114.5	179.7	127.8
	calc. *T*_1_	115.2	114.2	179.9	127.8
*δ* = C-Pt-X-Y [°]	exp.	129.9(1)	68.8(2)	17.8(5)	57.9(1)
	calc. *S*_0_	111.1	65.5	5.1	64.2
	calc. *T*_1_	107.7	101.9	−33.0	64.2

^a^ The X atom is the S, P, or C donor atom of the coligand, Y represents the following atom.

**Table 2:** Selected electrochemical data of the [Pt(dba)L] complexes (**1** to **4**) ^a^.

	Reduction	Reduction		Oxidation	Echem Gap
L =	*E*_1/2_ Red2	*E*_1/2_ Red1	ΔRed1−Red2	*E*_pa_ Ox1	ΔOx1−Red1
dmso (**1**)	−2.70 (irr)	−1.98 (irr)	0.72	0.93	2.91
PPh_3_ (**2**)	−2.70 (irr)	−1.96	0.74	0.75	2.71
CN*t*Bu (**3**)	−3.26	−1.89	1.37	0.80	2.69
Me_2_Imd (**4**)	−3.02 (irr)	−2.10	0.92	0.55	2.65

^a^ From cyclic voltammetry in *n*-Bu_4_NPF_6_/THF. Potentials in V vs. ferrocene/ferrocenium; half-wave potentials (*E*_1/2_) for reversible processes; anodic peak potentials *E*_pa_ for irreversible oxidations; cathodic peak potentials (*E*_pc_) for irreversible reductions (irr); accuracy of potentials: ±0.01 V.

**Table 3:** Experimental long-wavelength absorption maxima of the [Pt(dba)(L)] complexes (**1** to **4**) ^a^.

L =	*λ*_max_ (*ε*)	*λ*_max_ (*ε*)	*λ*_max_ (*ε*)	λ_max_ (*ε*)	*λ*_max_/eV	*λ*_cut-off_ ^b^/nm	*λ*_cut-off_ ^b^/eV
dmso (**1**)	292 (47.8)	340 (8.3)	409 (4.1)	525 (1.9)	2.36	566	2.19
PPh_3_ (**2**)	296 (50.2)	347 (12.7)	409 (6.2)	532 (2.1)	2.33	577	2.15
CN*t*Bu (**3**)	294 (27.0)	343 (5.2)	408 (2.4)	533 (1.0)	2.33	578	2.15
Me_2_Imd (**4**)	300 (24.8)	352 (8.9)	406 (3.2)	536 (1.3)	2.31	580	2.14

^a^ Measured in CH_2_Cl_2_, absorption maxima *λ*_max_ in nm, molar absorption coefficients ε in 10^3^ M^−1^ cm^−1^. ^b^ The *λ*_cut-off_ is defined as the wavelength of zero absorption and is obtained through extrapolation of the low-energy slope of the lowest energy absorption band.

**Table 4:** Photophysical properties of the [Pt(dba)(L)] complexes (**1** to **4**) and H_2_dba ^a^.

L =	*τ*_(air)_/ns	*τ*_(Ar)_/µs	*τ*_(77 K)_/µs	*Φ*_L (air)_ ± 0.02	*Φ*_L (Ar)_ ± 0.02	*Φ*_L (77 K)_ ± 0.02	*k*_r (77 K)_/ 10^4^ s^−1^
dmso (**1**)	*τ* = 336.0 ± 0.5	*τ* = 14.040 ± 0.013	*τ*_1_ = 27.9 ± 1.2 (24%) *τ*_2_ = 47.4 ± 0.4 (76%) *τ*_av_amp_ = 42.79 ± 0.06	<0.02	0.24	0.82	1.90 ± 0.05
PPh_3_ (**2**)	*τ*_1_ = 157.1 ± 0.3 (27%) *τ*_2_ = 4.1± 0.3 (73%) *τ*_av_amp_ = 46 ± 4	*τ* = 9.279 ± 0.008	*τ*_1_ = 40 ± 3 (14%) *τ*_2_ = 26.4 ± 0.4 (86%) *τ*_av_amp_ = 28.40 ± 0.03	<0.02	<0.02	0.90	3.16 ± 0.08
CN*t*Bu (**3**)	*τ*_1_ = 259.2 ± 0.7 (52%) *τ*_2_ = 6.9 ± 0.3 (48%) *τ*_av_amp_ = 139.0 ± 1.6	*τ* = 3.167 ± 0.002	*τ*_1_ = 38.4 ± 0.6 (14%) *τ*_2_ = 27.08 ± 0.12 (86%) *τ*_av_amp_ = 28.640 ± 0.016	0.04	0.11	0.80	2.80 ± 0.08
Me_2_Imd (**4**)	*τ*_1_ = 340 ± 20 (1%) *τ*_2_ = 7.0 ± 0.3 (24%) *τ*_3_ = 21.0 ± 1.2 (75%) *τ*_av_amp_ = 22.4 ± 0.6	*τ*_1_ = 2.13 ± 0.07 (2%) *τ*_2_ = 0.022 ± 0.001 (98%) *τ*_av_amp_ = 0.061 ± 0.004	*τ*_1_ = 60 ± 2 (2%) *τ*_2_ = 15.59 ± 0.05 (98%) *τ*_av_amp_ = 16.7 ± 0.04	<0.02	0.04	0.86	5.16 ± 0.13
H_2_dba	*τ*/10^−3^= 6.404 ± 0.009	*τ* = 6.81 ± 0.03	n.d.	0.42	0.42	0.53	n.d.

^a^ Photoluminescence quantum yields *Φ*_L_ (±0.02), lifetimes *τ*, radiative and non-radiative deactivation rate constants (*k*_r_ and *k*_nr_) under different conditions: air-equilibrated (air) or argon-purged samples (Ar) at 298 K or in frozen CH_2_Cl_2_:MeOH 1:1 glassy matrices at 77 K. For the bi-exponential decays, the amplitude-weighted average lifetimes (*τ*_av_amp_) [64] are shown. Raw time-resolved photoluminescence decays including the individual fitting components and their relative amplitudes (for biexponential decays) are available in the SM, Figures S16 to S29. Uncertainties for *k*_r_ were calculated as follows: $∆k_{r}=\left(\frac{∆\Phi_{L}}{\tau_{av}}\right)+\left(\frac{\Phi_{L}}{\tau_{av}{}^{2}}\;∆\tau_{av}\right)$ whereas for *k*_nr_ we used $∆k_{nr}=\left(\frac{∆\Phi_{L}}{\tau_{av}}\right)+\left(\frac{∆\tau_{av}}{\tau_{av}{}^{2}}\;\right)+\left(\frac{\Phi_{L}}{\tau_{av}{}^{2}}\;∆\tau_{av}\right)$. Uncertainties of *τ* are given by the decay fitting. n.d. = not determined, due to bad signal-to-noise ratios.

## Data Availability

Available from the authors on request.

## Supplementary Materials

The following supporting information can be downloaded at: https://www.mdpi.com/article/10.3390/molecules27228054/s1, Figure S1: Crystal structure of [Pt(dba)(CN*t*Bu)] viewed along the crystallographic *c* axis, the stacking of two molecules in the unit cell, and the complex molecule. Figure S2: Crystal structure of [Pt(dba)(PPh_3_)] viewed along the crystallographic *b* axis, the stacking of two molecules in the unit cell, and the complex molecule. Figure S3: Crystal structure of [Pt(dba)(DMSO)] viewed along the crystallographic *a* axis, the stacking of two molecules in the unit cell, and the complex molecule. Figure S4: Crystal structure of [Pt(dba)(Me_2_Imd)] viewed along the crystallographic *b* axis (left). Figure S5: DFT-optimized *S*_0_ ground state geometries of the complex [Pt(dba)(L)] with L = DMSO, PPh_3_, Me_2_Imd, and CN*t*Bu. Figure S6: DFT-optimized *T*_1_ excited state geometries of the complex [Pt(dba)(L)] with L = DMSO, PPh_3_, Me_2_Imd, and CN*t*Bu. Figure S7: Cyclic voltammogramms of [Pt(dba)(dmso)] and [Pt(db(ph)a)(dmso)] in 0.1 M *n*-Bu_4_NPF_6_/THF. Figure S8: DFT-calculated energies and composition of selected frontier molecular orbitals for [Pt(dba)(L)] (L = dmso, PPh_3_, Me_2_Imd, and CN*t*Bu) at the optimized ground state *S*_0_ geometry. Figure S9: UV-vis absorption spectra of [Pt(dba)(L)] (L = dmso, PPh_3_, CN*t*Bu and Me_2_Imd) in CH_2_Cl_2_. Figure S10: Normalized photoluminescence spectra of the [Pt(dba)L] complexes in CH_2_Cl_2_ at 298 K. Figure S11: Normalized excitation (dotted line) and emission (continuous line) spectra of [Pt(dba)(dmso)] in a CH_2_Cl_2_/MeOH 1:1 glassy matrix at 77 K. Figure S12: Normalized excitation (dotted line) and emission (continuous line) spectra of [Pt(dba)(PPh_3_)] in a CH_2_Cl_2_/MeOH 1:1 glassy matrix at 77 K. Figure S13: Normalized excitation (dotted line) and emission (continuous line) spectra of [Pt(dba)(CN*t*Bu)] in a CH_2_Cl_2_/MeOH 1:1 glassy matrix at 77 K. Figure S14: Normalized excitation (dotted line) and emission (continuous line) spectra of [Pt(dba)(Me_2_Imd)] in a CH_2_Cl_2_/MeOH 1:1 glassy matrix at 77 K. Figure S15: Left: Time-resolved photoluminescence of [Pt(dba)(dmso)] in an air-equilibrated CH_2_Cl_2_ solution at 298 K (*c* = 10^−5^ M), including the residuals (λ_ex_ = 376 nm, λ_em_ = 600 nm). Right: Fitting parameters including pre-exponential factors and confidence limits. Figure S16: Left: Time-resolved photoluminescence of [Pt(dba)(dmso)] in an Ar-purged CH_2_Cl_2_ solution at 298 K (*c* = 10^−5^ M), including the residuals (λ_ex_ = 376 nm, λ_em_ = 600 nm). Right: Fitting parameters including pre-exponential factors and confidence limits. Figure S17: Left: Time-resolved photoluminescence of [Pt(dba)(dmso)] in a frozen CH_2_Cl_2_/MeOH glassy matrix at 77 K (*c* = 10^−5^ M), including the residuals (λ_ex_ = 376 nm, λ_em_ = 600 nm). Right: Fitting parameters including pre-exponential factors and confidence limits. Figure S18: Left: Time-resolved photoluminescence of [Pt(dba)(PPh_3_)] in an air-equilibrated CH_2_Cl_2_ solution at 298 K (*c* = 10^−5^ M), including the residuals (λ_ex_ = 376 nm, λ_em_ = 600 nm). Right: Fitting parameters including pre-exponential factors and confidence limits. Figure S19: Left: Time-resolved photoluminescence of [Pt(dba)(PPh_3_)] in an Ar-purged CH_2_Cl_2_ solution at 298 K (*c* = 10^−5^ M), including the residuals (λ_ex_ = 376 nm, λ_em_ = 600 nm). Right: Fitting parameters including pre-exponential factors and confidence limits. Figure S20: Left: Time-resolved photoluminescence of [Pt(dba)(PPh_3_)] in a frozen CH_2_Cl_2_/MeOH glassy matrix at 77 K (*c* = 10^−5^ M), including the residuals (λ_ex_ = 376 nm, λ_em_ = 600 nm). Right: Fitting parameters including pre-exponential factors and confidence limits. Figure S21: Left: Time-resolved photoluminescence of [Pt(dba)(CN*t*Bu)] in an air-equilibrated CH_2_Cl_2_ solution at 298 K (*c* = 10^−5^ M), including the residuals (λ_ex_ = 376 nm, λ_em_ = 600 nm). Right: Fitting parameters including pre-exponential factors and confidence limits. Figure S22: Left: Time-resolved photoluminescence of [Pt(dba)(CN*t*Bu)] in an Ar-purged CH_2_Cl_2_ solution at 298 K (*c* = 10^−5^ M), including the residuals (λ_ex_ = 376 nm, λ_em_ = 600 nm). Right: Fitting parameters including pre-exponential factors and confidence limits. Figure S23: Left: Time-resolved photoluminescence of [Pt(dba)(CN*t*Bu)] in a frozen CH_2_Cl_2_/MeOH glassy matrix at 77 K (*c* = 10^−5^ M), including the residuals (λ_ex_ = 376 nm, λ_em_ = 600 nm). Right: Fitting parameters including pre-exponential factors and confidence limits. Figure S24: Left: Time-resolved photoluminescence of [Pt(dba)(Me_2_Imd)] in an air-equilibrated CH_2_Cl_2_ solution at 298 K (*c* = 10^−5^ M), including the residuals (λ_ex_ = 376 nm, λ_em_ = 600 nm). Right: Fitting parameters including pre-exponential factors and confidence limits. Figure S25: Left: Time-resolved photoluminescence of [Pt(dba)(Me_2_Imd)] in an Ar-purged CH_2_Cl_2_ solution at 298 K (*c* = 10^−5^ M), including the residuals (λ_ex_ = 376 nm, λ_em_ = 600 nm). Right: Fitting parameters including pre-exponential factors and confidence limits. Figure S26: Left: Time-resolved photoluminescence of [Pt(dba)(Me_2_Imd)] in a frozen CH_2_Cl_2_/MeOH glassy matrix at 77 K (*c* = 10^−5^ M), including the residuals (λ_ex_ = 376 nm, λ_em_ = 600 nm). Right: Fitting parameters including pre-exponential factors and confidence limits. Figure S27: UV-vis absorption spectra of [Pt(dba)(dmso)] in THF/*n-*Bu_4_NPF_6_, recorded before and after cathodic reduction and before and after anodic oxidation. Figure S28: UV-vis absorption spectra of [Pt(dba)(PPh_3_)] in THF/*n-*Bu_4_NPF_6_, recorded before and after cathodic reduction and before and after anodic oxidation. Figure S29: UV-vis absorption spectra of [Pt(dba)(CN*t*Bu)] in THF/*n-*Bu_4_NPF_6_, recorded before and after cathodic reduction and before and after anodic oxidation. Figure S30: UV-vis absorption spectra of [Pt(dba)(Me_2_Imd)] in THF/*n-*Bu_4_NPF_6_, recorded before and after cathodic reduction and before and after anodic oxidation. Table S1: Selected structure solution and refinement data for [Pt(dba)(Me_2_Imd)], [Pt(dba)(CN*t*Bu)], [Pt(dba)(PPh_3_)] and [Pt(dba)(dmso)]. Table S2: Selected experimental structural data of [Pt(dba)(Me_2_Imd)], [Pt(dba)(CN*t*Bu)], [Pt(dba)(PPh_3_)], and [Pt(dba)(dmso)]. Table S3: Electrochemical data of the Pt(II) complexes. Table S4: Experimental long-wavelength absorption maxima of the Pt(II) complexes. Table S5: Selected absorption maxima of the oxidized, parent, and reduced [Pt(dba)(L)]^+/0/−^ complexes. Figure S31: 600 MHz ^1^H NMR spectra of [Pt(dba)(PPh_3_)] in CD_2_Cl_2_. Figure S32: 600 MHz ^31^P NMR spectrum of [Pt(dba)(PPh_3_)] in CD_2_Cl_2_. Figure S33: 600 MHz H,H COSY NMR spectrum of [Pt(dba)(PPh_3_)] in CD_2_Cl_2_. Figure S34: 600 MHz H,H NOESY NMR spectrum of [Pt(dba)(PPh_3_)] in CD_2_Cl_2_. Figure S35: 600 MHz ^1^H NMR spectrum of [Pt(dba)(CN*t*Bu)] in CD_2_Cl_2_. Figure S36: 300 MHz ^13^C-135-DEPTQ-[^1^H] NMR spectra of [Pt(dba)(CN*t*Bu)] in CD_2_Cl_2_. Figure S37: 600 MHz H,H COSY NMR spectrum of [Pt(dba)(CN*t*Bu)] in CD_2_Cl_2_. Figure S38: 600 MHz ^1^H NMR spectrum of [Pt(dba)(Me_2_Imd)] in CD_2_Cl_2_. Figure S39: 300 MHz ^13^C-135-DEPTQ-[^1^H] NMR spectra of [Pt(dba)(Me_2_Imd)] in CD_2_Cl_2_. Figure S40: 600 MHz H,H COSY NMR spectrum of [Pt(dba)(Me_2_Imd)] in CD_2_Cl_2_. Figure S41: 300 MHz ^1^H-^195^Pt-HMBC NMR spectrum of [Pt(dba)(Me_2_Imd)] in CD_2_Cl_2_. Figure S42: ESI-MS(+) of [Pt(dba)(PPh_3_)]. Figure S43: ESI-MS(+) of [Pt(dba)(CN*t*Bu)]. Figure S44: EI-MS(+) of [Pt(dba)(Me_2_Imd)].
