# Supplementary material for: Triplet Emitting C^N^C Cyclometalated Dibenzo[c,h]Acridine Pt(II) Complexes
Source: Molecules. 2022 Nov 19;27(22):8054. doi: 10.3390/molecules27228054 (PMC9697690; doi:10.3390/molecules27228054)
Supplement: Supplementary file 1 [file molecules-27-08054-s001.zip › molecules-2028679-supplementary.pdf]

Supplementary Materials

for

Triplet emitting C^N^C cyclometalated dibenzo[c,h]acridine Pt(II) complexes

Part I

Joshua Nicolas Friedel, Maren Krause, Rose Jordan, Iván Maisuls, Dana Brünink, Dominik Schwab, Nikos L. Doltsinis,* Cristian A. Strassert,* Axel Klein*

Contents

Supplementary Figures

Figure S1. Crystal structure of [Pt(dba)(CN*t*Bu)] viewed along the crystallographic *c* axis, the stacking of two molecules in the unit cell, and view on the complex molecule.

Figure S2. Crystal structure of [Pt(dba)(PPh_3)] viewed along the crystallographic *b* axis, the stacking of two molecules in the unit cell, and view on the complex molecule.

Figure S3. Crystal structure of [Pt(dba)(DMSO)] viewed along the crystallographic *a* axis, the stacking of two molecules in the unit cell, and view on the complex molecule.

Figure S4. Crystal structure of [Pt(dba)(Me_2Imd)] viewed along the crystallographic *a* and *b* axis and view on the complex molecule.

Figure S5. DFT-optimized S_0 ground state geometries of the complex [Pt(dba)(L)] with L = DMSO, PPh_3, Me_2Imd, and CN*t*Bu.

Figure S6. DFT-optimized T_1 excited state geometries of the complex [Pt(dba)(L)] with L = DMSO, PPh_3, Me_2Imd, and CN*t*Bu.

Figure S7. Cyclic voltammogramms of [Pt(dba)(dmso)] and [Pt(db(ph)a)(dmso)] in 0.1 M *n*-Bu_4NPF_6/THF.

Figure S8. DFT-calculated energies and composition of selected frontier molecular orbitals for [Pt(dba)(L)] (L = dmso, PPh_3, Me_2Imd, and CN*t*Bu) at the optimized ground state S_0 geometry.

Figure S9. UV-vis absorption spectra of [Pt(dba)(L)] (L = dmso, PPh_3, CN*t*Bu and Me_2Imd) in CH_2Cl_2.

Figure S10. Normalized photoluminescence spectra of the [Pt(dba)L] complexes in CH_2Cl_2 at 298 K.

Figure S11. Normalized excitation (dotted line) and emission (continuous line) spectra of [Pt(dba)(dmso)] in a CH_2Cl_2/MeOH 1:1 glassy matrix at 77 K.

Figure S12. Normalized excitation (dotted line) and emission (continuous line) spectra of [Pt(dba)(PPh_3)] in a CH_2Cl_2/MeOH 1:1 glassy matrix at 77 K.

Figure S13. Normalized excitation (dotted line) and emission (continuous line) spectra of [Pt(dba)(CN*t*Bu)] in a CH_2Cl_2/MeOH 1:1 glassy matrix at 77 K.

Figure S14. Normalized excitation (dotted line) and emission (continuous line) spectra of [Pt(dba)(Me_2Imd)] in a CH_2Cl_2/MeOH 1:1 glassy matrix at 77 K.

Figure S15. Left: Time-resolved photoluminescence of [Pt(dba)(dmso)] in an air-equilibrated CH_2Cl_2 solution at 298 K ($c = 10^{-5}$ M), including the residuals (λ_{ex} = 376 nm, λ_{em} = 600 nm). Right: Fitting parameters including pre-exponential factors and confidence limits.

Figure S16. Left: Time-resolved photoluminescence of [Pt(dba)(dmso)] in an Ar-purged CH_2Cl_2 solution at 298 K ($c = 10^{-5}$ M), including the residuals (λ_{ex} = 376 nm, λ_{em} = 600 nm). Right: Fitting parameters including pre-exponential factors and confidence limits.

Figure S17. Left: Time-resolved photoluminescence of [Pt(dba)(dmso)] in a frozen CH_2Cl_2/MeOH glassy matrix at 77K ($c = 10^{-5}$ M), including the residuals (λ_{ex} = 376 nm, λ_{em} = 600 nm). Right: Fitting parameters including pre-exponential factors and confidence limits.

Figure S18. Left: Time-resolved photoluminescence of [Pt(dba)(PPh_3)] in an air-equilibrated CH_2Cl_2 solution at 298 K ($c = 10^{-5}$ M), including the residuals (λ_{ex} = 376 nm, λ_{em} = 600 nm). Right: Fitting parameters including pre-exponential factors and confidence limits.

Figure S19. Left: Time-resolved photoluminescence of [Pt(dba)(PPh_3)] in an Ar-purged CH_2Cl_2 solution at 298 K (c = 10^{-5} M), including the residuals (λ_{ex} = 376 nm, λ_{em} = 600 nm). Right: Fitting parameters including pre-exponential factors and confidence limits.
Figure S20. Left: Time-resolved photoluminescence of [Pt(dba)(PPh_3)] in a frozen CH_2Cl_2/MeOH glassy matrix at 77 K (c = 10^{-5} M), including the residuals (λ_{ex} = 376 nm, λ_{em} = 600 nm). Right: Fitting parameters including pre-exponential factors and confidence limits.
Figure S21. Left: Time-resolved photoluminescence of [Pt(dba)(CN*t*Bu)] in an air-equilibrated CH_2Cl_2 solution at 298 K (c = 10^{-5} M), including the residuals (λ_{ex} = 376 nm, λ_{em} = 600 nm). Right: Fitting parameters including pre-exponential factors and confidence limits.
Figure S22. Left: Time-resolved photoluminescence of [Pt(dba)(CN*t*Bu)] in an Ar-purged CH_2Cl_2 solution at 298 K (c = 10^{-5} M), including the residuals (λ_{ex} = 376 nm, λ_{em} = 600 nm). Right: Fitting parameters including pre-exponential factors and confidence limits.
Figure S23. Left: Time-resolved photoluminescence of [Pt(dba)(CN*t*Bu)] in a frozen CH_2Cl_2/MeOH glassy matrix at 77 K (c = 10^{-5} M), including the residuals (λ_{ex} = 376 nm, λ_{em} = 600 nm). Right: Fitting parameters including pre-exponential factors and confidence limits.
Figure S24. Left: Time-resolved photoluminescence of [Pt(dba)(Me_2Imd)] in an air-equilibrated CH_2Cl_2 solution at 298 K (c = 10^{-5} M), including the residuals (λ_{ex} = 376 nm, λ_{em} = 600 nm). Right: Fitting parameters including pre-exponential factors and confidence limits.
Figure S25. Left: Time-resolved photoluminescence of [Pt(dba)(Me_2Imd)] in an Ar-purged CH_2Cl_2 solution at 298 K (c = 10^{-5} M), including the residuals (λ_{ex} = 376 nm, λ_{em} = 600 nm). Right: Fitting parameters including pre-exponential factors and confidence limits.
Figure S26. Left: Time-resolved photoluminescence of [Pt(dba)(Me_2Imd)] in a frozen CH_2Cl_2/MeOH glassy matrix at 77 K (c = 10^{-5} M), including the residuals (λ_{ex} = 376 nm, λ_{em} = 600 nm). Right: Fitting parameters including pre-exponential factors and confidence limits.
Figure S27. UV-vis absorption spectra of [Pt(dba)(dmso)] in THF/*n*-Bu_4NPF_6, recorded before and after cathodic reduction and before and after anodic oxidation.
Figure S28. UV-vis absorption spectra of [Pt(dba)(PPh_3)] in THF/*n*-Bu_4NPF_6, recorded before and after cathodic reduction and before and after anodic oxidation.
Figure S29. UV-vis absorption spectra of [Pt(dba)(CN*t*Bu)] in THF/*n*-Bu_4NPF_6, recorded before and after cathodic reduction and before and after anodic oxidation.
Figure S30. UV-vis absorption spectra of [Pt(dba)(Me_2Imd)] in THF/*n*-Bu_4NPF_6, recorded before and after cathodic reduction and before and after anodic oxidation.

Supplementary Tables

Table S1. Selected structure solution and refinement data for [Pt(dba)(Me_2Imd)], [Pt(dba)(CN*t*Bu)], [Pt(dba)(PPh_3)] and [Pt(dba)(dmso)].
Table S2. Selected experimental structural data of [Pt(dba)(Me_2Imd)], [Pt(dba)(CN*t*Bu)], [Pt(dba)(PPh_3)], and [Pt(dba)(dmso)].
Table S3. Electrochemical data of the Pt complexes.
Table S4. Experimental long-wavelength absorption maxima of the [Pt(dba)(L)] complexes.
Table S5. Selected absorption maxima of the oxidized, parent, and reduced $[Pt(dba)(L)]^{+/0/-}$ complexes.

Supplementary Figures

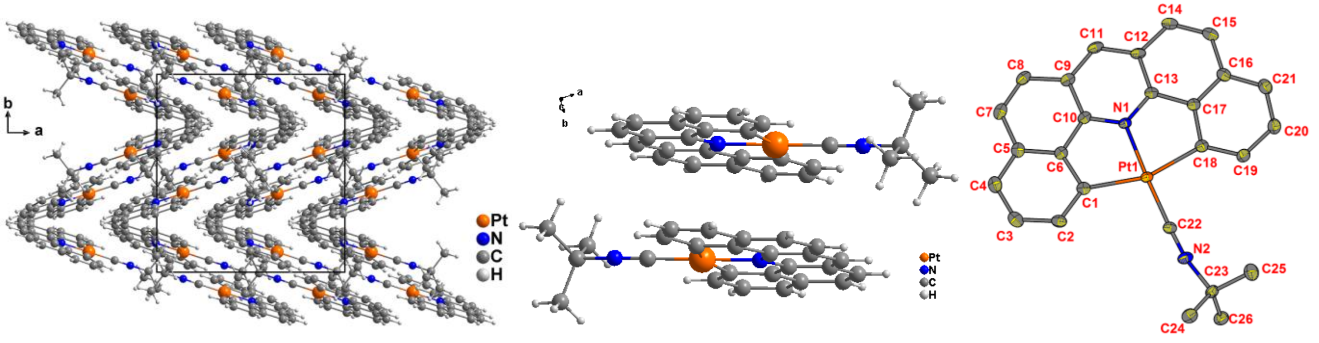

Figure S1. Crystal structure of [Pt(dba)(CN*t*Bu)] viewed along the crystallographic *c* axis (left), the stacking of two molecules in the unit cell (center) and view on the complex molecule (right) with elipsoids at 50% probability with numbering, H atoms were omitted for clarity.

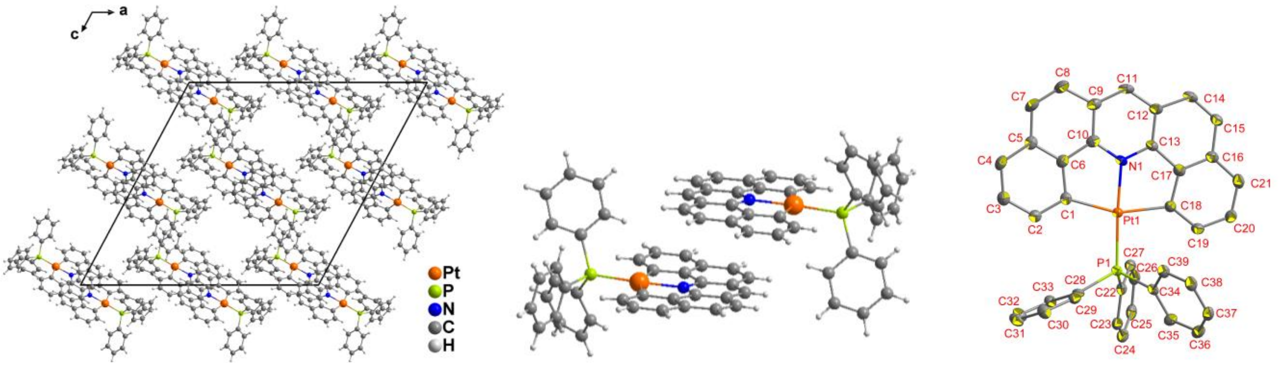

Figure S2. Crystal structure of [Pt(dba)(PPh_3)] viewed along the crystallographic *b* axis (left), the stacking of two molecules in the unit cell (center) and view on the complex molecule (right) with elipsoids at 50% probability with numbering, H atoms were omitted for clarity.

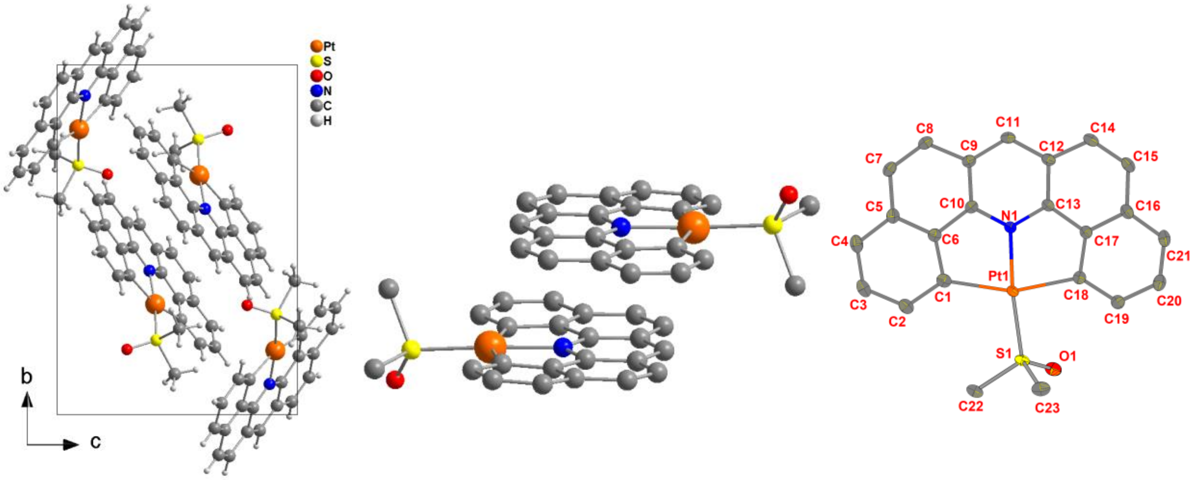

Figure S3. Crystal structure of [Pt(dba)(DMSO)] viewed along the crystallographic *a* axis (left), the stacking of two molecules in the unit cell (center) and view on the complex molecule (right) with elipsoids at 50% probability with numbering, H atoms were omitted for clarity. The centroid···centroid distance for the stacking is 3.62 Å with an offset of 1.25 Å.

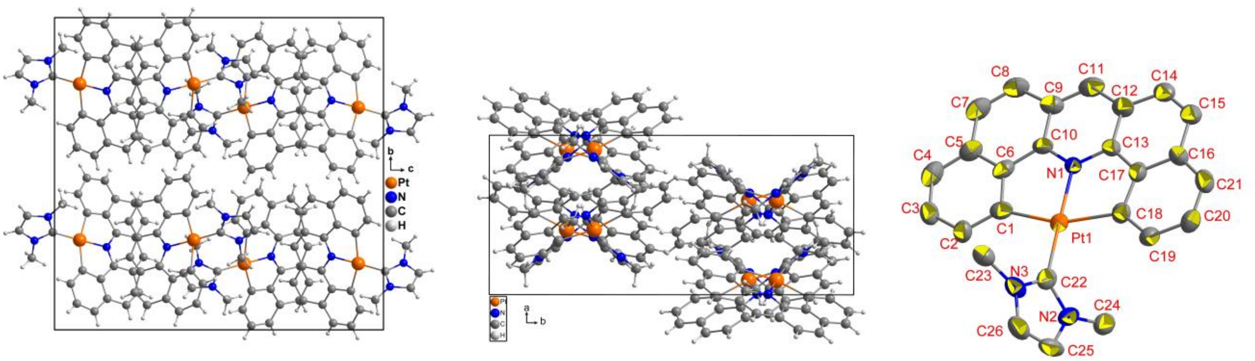

Figure S4. Crystal structures of [Pt(dba)(Me_2Imd)] viewed along the crystallographic *a* (left) and *c* axis (center) and view on the complex molecule (right) with elipsoids at 50% probability with numbering, H atoms were omitted for clarity.

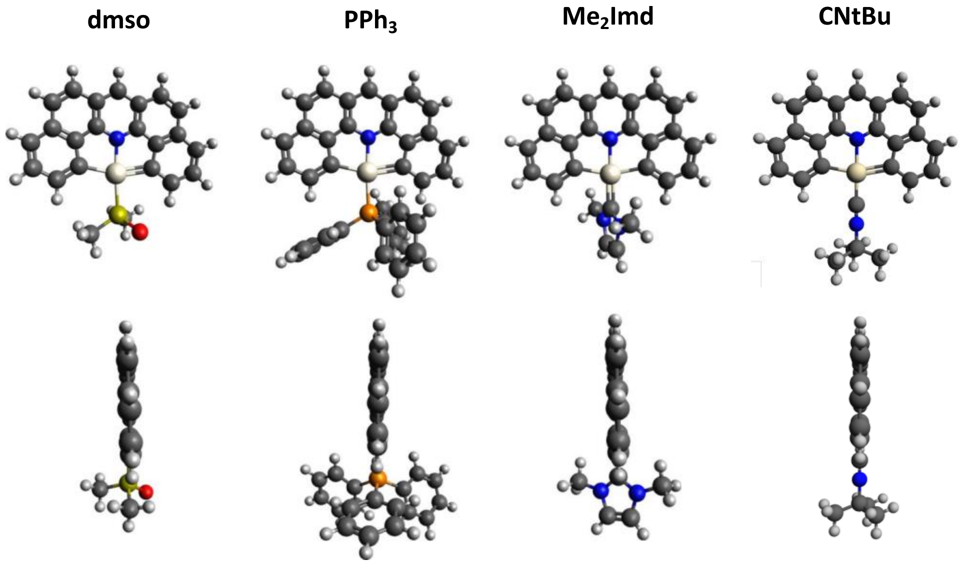

Figure S5. DFT-optimized S_0 ground state geometries of the complex [Pt(dba)(L)] with L= DMSO, PPh_3, Me_2Imd, CN*t*Bu in CH_2Cl_2 in front and side view.

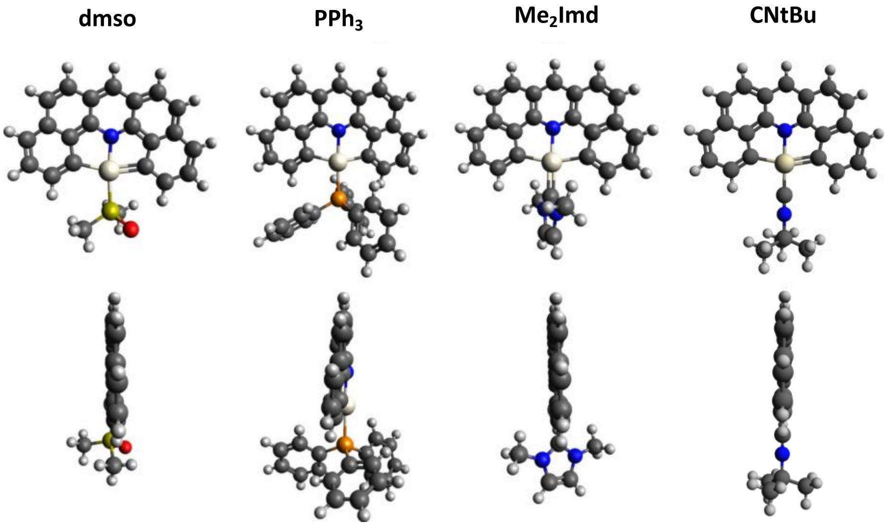

Figure S6. DFT-optimized T_1 excited state geometries of the complex [Pt(dba)(L)] with L= DMSO, PPh_3, Me_2Imd, CN*t*Bu in CH_2Cl_2 in front and side view.

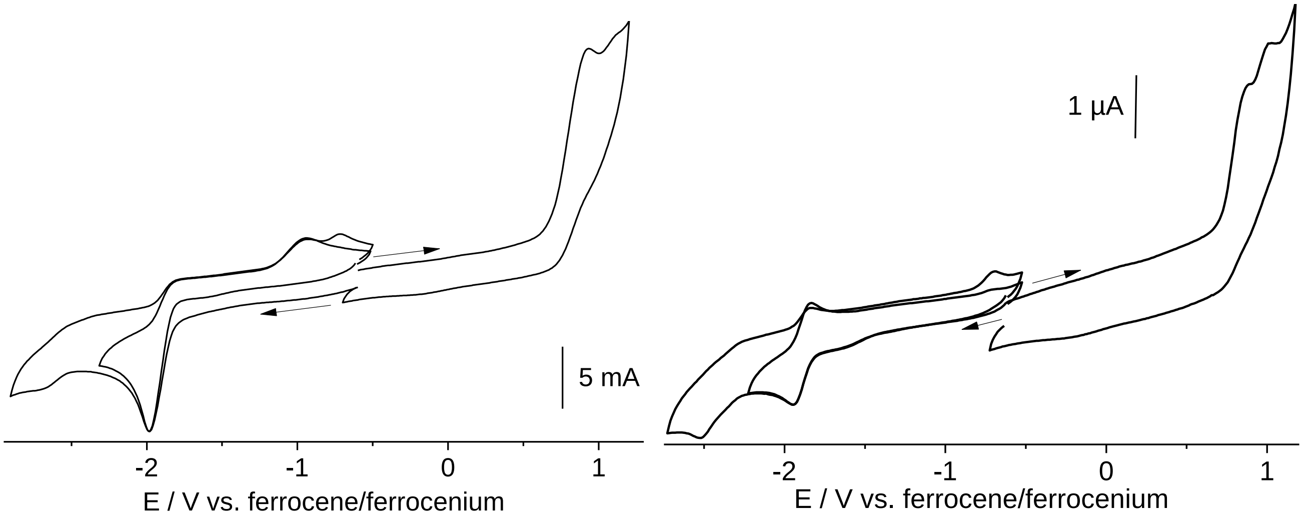

Figure S7. Cyclic voltammogramms of [Pt(dba)(dmso)] (left) and [Pt(db(ph)a)(dmso)] (right) in 0.1 M *n*-Bu_4NPF_6/THF.

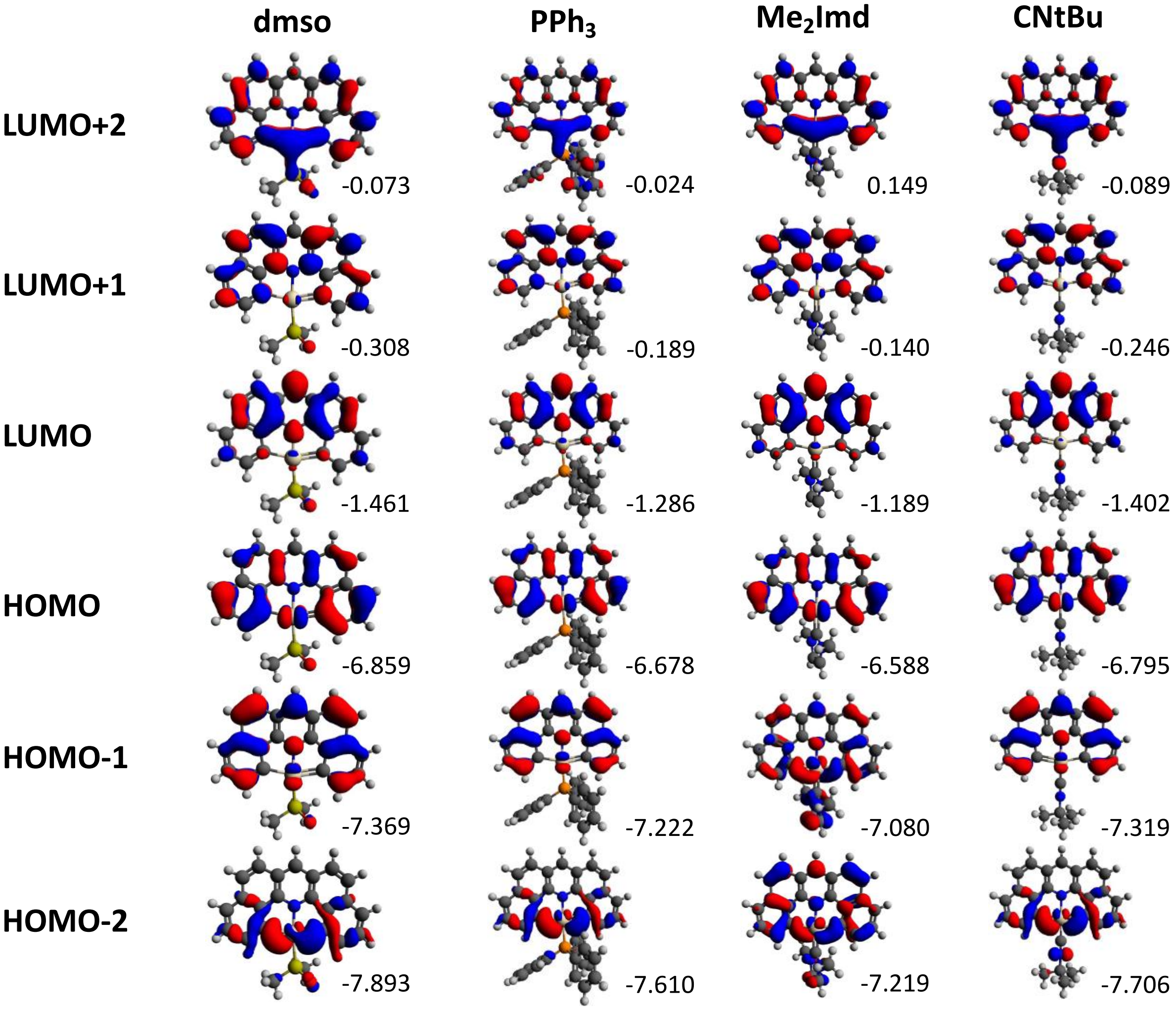

Figure S8. DFT-calculated energies and composition of selected frontier molecular orbitals for [Pt(dba)(L)] (L = dmso, PPh_3, Me_2Imd, and CN*t*Bu) at the optimized ground state S_0 geometry.

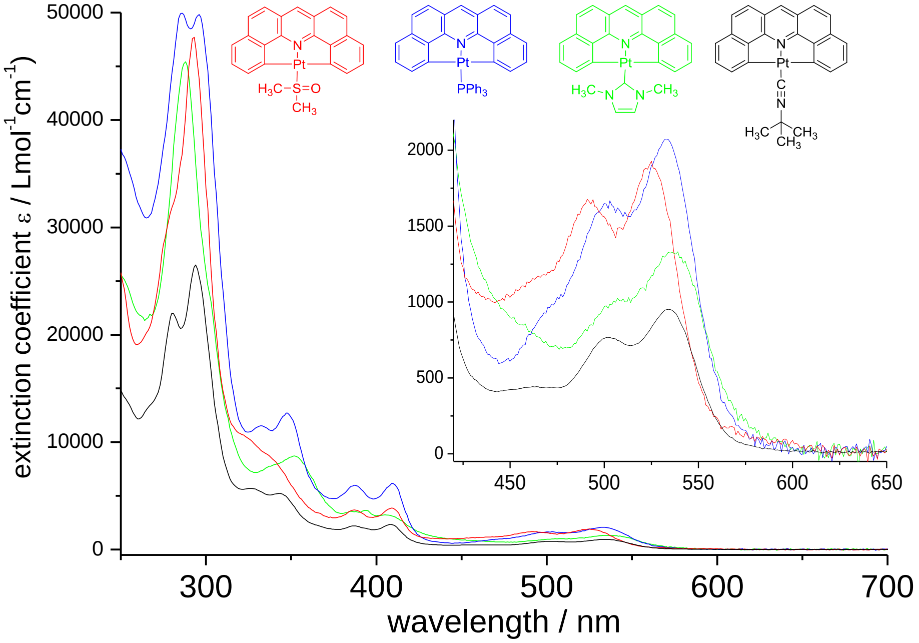

Figure S9. UV-vis absorption spectra of [Pt(dba)(L)] (L = dmso, PPh_3, CN*t*Bu and Me_2Imd) in CH_2Cl_2.

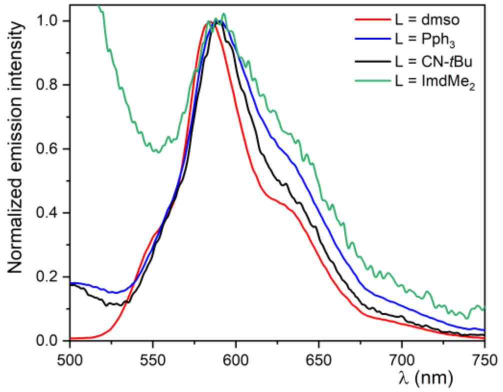

Figure S10. Normalized photoluminescence spectra of the [Pt(dba)L] complexes in CH_2Cl_2 at 298 K.

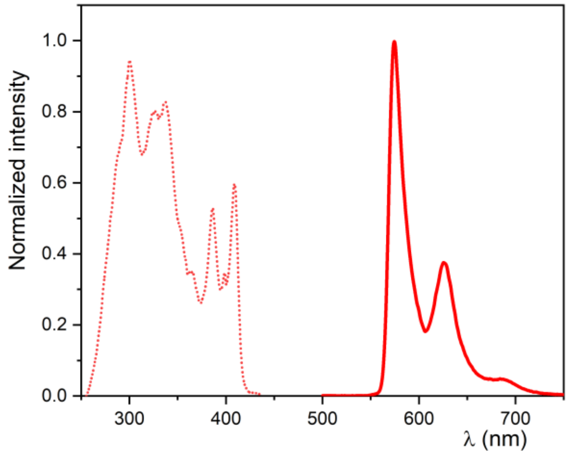

Figure S11. Normalized excitation (dotted line) and emission (continuous line) spectra of [Pt(dba)(dmso)] in a CH_2Cl_2/MeOH 1:1 glassy matrix at 77 K.

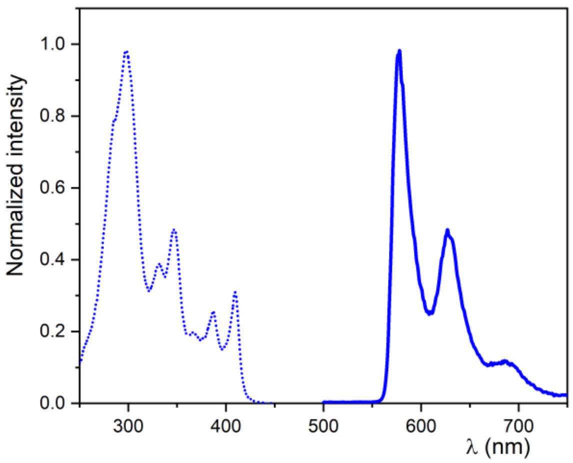

Figure S12. Normalized excitation (dotted line) and emission (continuous line) spectra of [Pt(dba)(PPh_3)] in a CH_2Cl_2/MeOH 1:1 glassy matrix at 77 K.

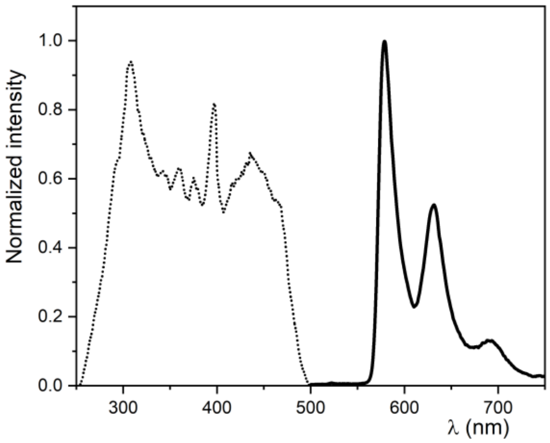

Figure S13. Normalized excitation (dotted line) and emission (continuous line) spectra of [Pt(dba)(CN*t*Bu)] in a CH_2Cl_2/MeOH 1:1 glassy matrix at 77 K.

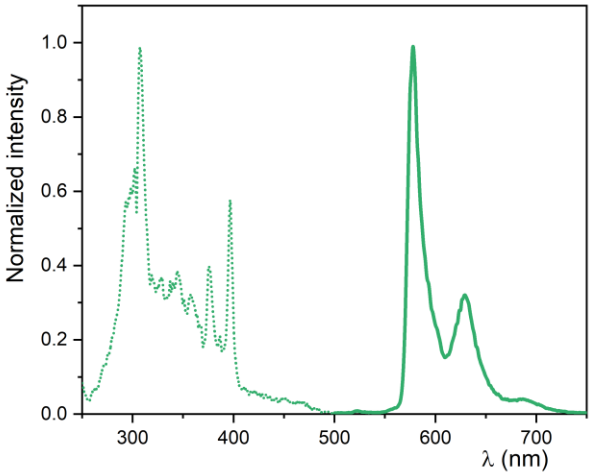

Figure S14. Normalized excitation (dotted line) and emission (continuous line) spectra of [Pt(dba)(Me_2Imd)] in a CH_2Cl_2/MeOH 1:1 glassy matrix at 77 K.

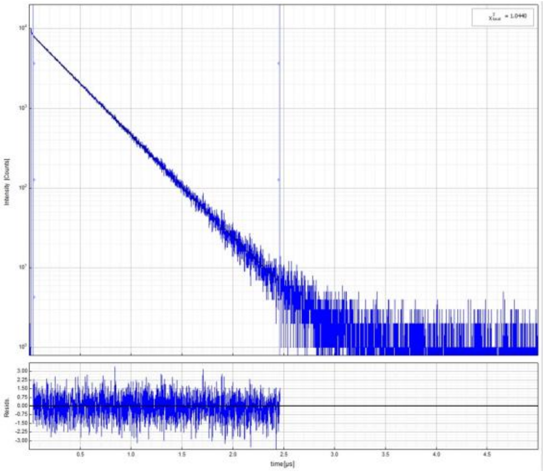

Parameter	Value	Error
A_1[kCnts/Chnl]	8.107	± 0.013
τ_1[ns]	336.00	± 0.53
I_1[kCnts]	2724.1	± 3.8
$A_{Rel\,1}$[%]	100.0000	± 0.0000
$I_{Rel\,1}$[%]	100.0000	± 0.0000
$Bkgr_{Dec}$[kCnts]	0.0006	± 0.0003
$\tau_{Av\,Int}$[ns]	336.00	± 0.53
$\tau_{Av\,Amp}$[ns]	336.00	± 0.53

Figure S15. Left: Time-resolved photoluminescence of [Pt(dba)(dmso)] in an air-equilibrated CH_2Cl_2 solution at 298 K ($c = 10^{-5}$ M), including the residuals (λ_{ex} = 376 nm, λ_{em} = 600 nm). Right: Fitting parameters including pre-exponential factors and confidence limits.

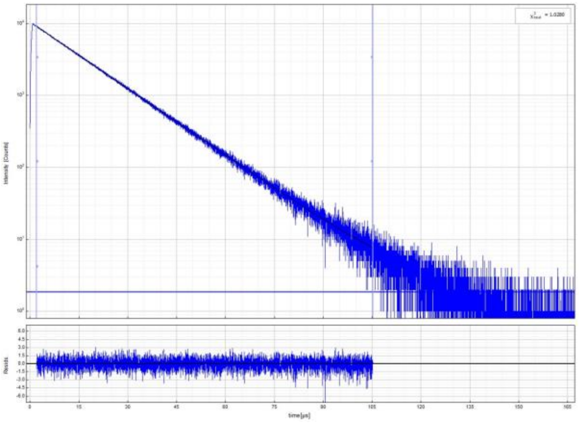

Parameter	Value	Error
A_1[kCnts/Chnl]	9.0799	± 0.0072
τ_1[ns]	14040	± 13
I_1[kCnts]	7967.7	± 5.7
$A_{Rel\,1}$[%]	100.0000	± 0.0000
$I_{Rel\,1}$[%]	100.0000	± 0.0000
$Bkgr_{Dec}$[kCnts]	0.0018	± 0.0002
$\tau_{Av\,Int}$[ns]	14040	± 13
$\tau_{Av\,Amp}$[ns]	14040	± 13

Figure S16. Left: Time-resolved photoluminescence of [Pt(dba)(dmso)] in an Ar-purged CH_2Cl_2 solution at 298 K ($c = 10^{-5}$ M), including the residuals (λ_{ex} = 376 nm, λ_{em} = 600 nm). Right: Fitting parameters including pre-exponential factors and confidence limits.

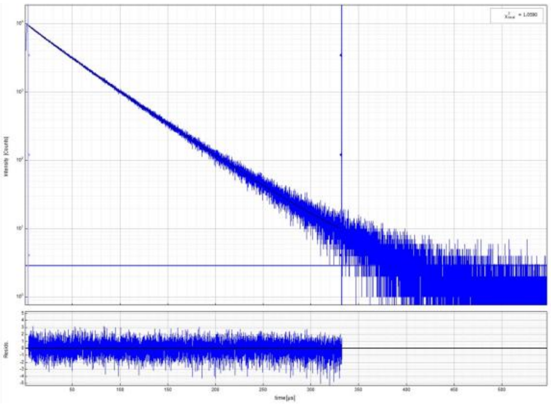

Parameter	Value	Error
A_1[kCnts/Chnl]	2,23	± 0,24
τ_1[ns]	27970	± 1161
I_1[kCnts]	1951	± 275
$A_{Rel\,1}$[%]	23,8	± 2,6
$I_{Rel\,1}$[%]	15,5	± 2,2
A_2[kCnts/Chnl]	7,15	± 0,24
τ_2[ns]	47415	± 373
I_2[kCnts]	10598	± 267
$A_{Rel\,2}$[%]	76,2	± 2,6
$I_{Rel\,2}$[%]	84,5	± 2,2
$Bkgr_{Dec}$[kCnts]	0,0029	± 0,0004
$\tau_{Av\,Int}$[ns]	44392	± 42
$\tau_{Av\,Amp}$[ns]	42790	± 58

Figure S17. Left: Time-resolved photoluminescence of [Pt(dba)(dmso)] in a frozen CH_2Cl_2/MeOH glassy matrix at 77 K ($c = 10^{-5}$ M), including the residuals (λ_{ex} = 376 nm, λ_{em} = 600 nm). Right: Fitting parameters including pre-exponential factors and confidence limits.

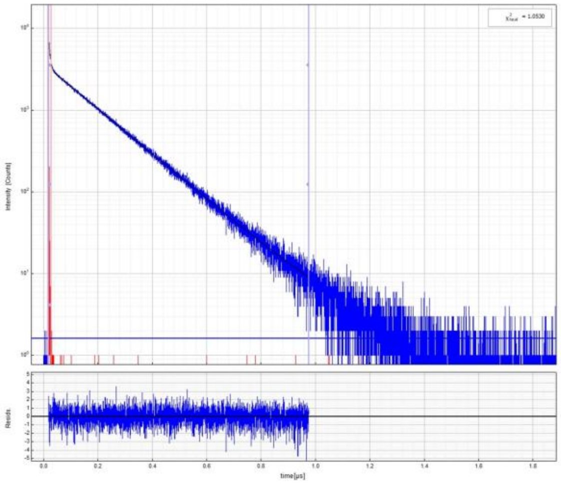

Parameter	Value	Error
A_1[kCnts/Chnl]	3.62	± 0.19
τ_1[ns]	157.11	± 0.32
I_1[kCnts]	2277	± 112
$A_{Rel\,1}$[%]	27.4	± 2.2
$I_{Rel\,1}$[%]	93.57	± 0.28
A_2[kCnts/Chnl]	9.6	± 1.1
τ_2[ns]	4.08	± 0.32
I_2[kCnts]	156.4	± 6.8
$A_{Rel\,2}$[%]	72.6	± 2.2
$I_{Rel\,2}$[%]	6.43	± 0.28
$Bkgr_{Dec}$[kCnts]	0.0016	± 0.0004
$Bkgr_{IRF}$[Cnts/Chnl]	-42	± 16
$Shift_{IRF}$[ps]	17	± 18
A_{Scat}[kCnts]	-13	± 25
$\tau_{Av\,Int}$[ns]	147.28	± 0.57
$\tau_{Av\,Amp}$[ns]	46.1	± 3.7

Figure S18. Left: Time-resolved photoluminescence of [Pt(dba)(PPh_3)] in an air-equilibrated CH_2Cl_2 solution at 298 K (c = 10^{-5} M), including the residuals (λ_{ex} = 376 nm, λ_{em} = 600 nm). Right: Fitting parameters including pre-exponential factors and confidence limits.

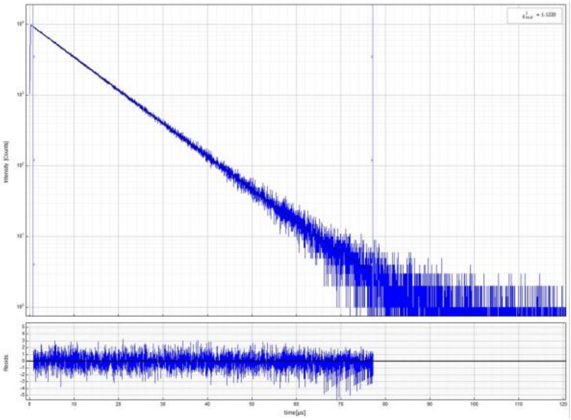

Parameter	Value	Error
A_1[kCnts/Chnl]	9.265	± 0.011
τ_1[ns]	9279.2	± 7.7
I_1[kCnts]	5373.3	± 3.2
$A_{Rel\,1}$[%]	100.0000	± 0.0000
$I_{Rel\,1}$[%]	100.0000	± 0.0000
$Bkgr_{Dec}$[kCnts]	0.0007	± 0.0002
$\tau_{Av\,Int}$[ns]	9279.2	± 7.7
$\tau_{Av\,Amp}$[ns]	9279.2	± 7.7

Figure S19. Left: Time-resolved photoluminescence of [Pt(dba)(PPh_3)] in an Ar-purged CH_2Cl_2 solution at 298 K (c = 10^{-5} M), including the residuals (λ_{ex} = 376 nm, λ_{em} = 600 nm). Right: Fitting parameters including pre-exponential factors and confidence limits.

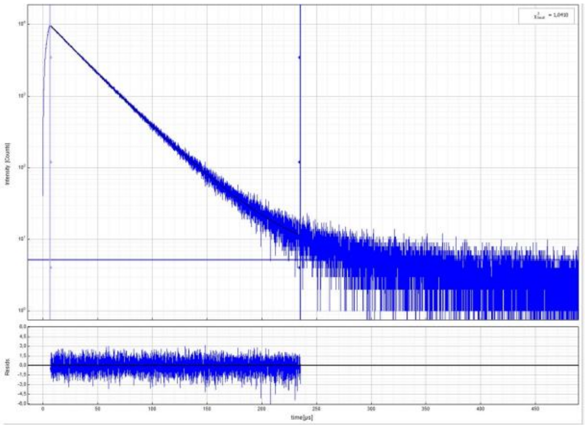

Parameter	Value	Error
A_1[kCnts/Chnl]	1,36	± 0,49
τ_1[ns]	40318	± 3479
I_1[kCnts]	1710	± 514
$A_{Rel\,1}$[%]	14,1	± 5,1
$I_{Rel\,1}$[%]	20,0	± 6,0
A_2[kCnts/Chnl]	8,30	± 0,48
τ_2[ns]	26447	± 438
I_2[kCnts]	6859	± 516
$A_{Rel\,2}$[%]	85,9	± 5,1
$I_{Rel\,2}$[%]	80,0	± 6,0
$Bkgr_{Dec}$[kCnts]	0,0052	± 0,0007
$\tau_{Av\,Int}$[ns]	29214	± 70
$\tau_{Av\,Amp}$[ns]	28396	± 34

Figure S20. Left: Time-resolved photoluminescence of [Pt(dba)(PPh_3)] in a frozen CH_2Cl_2/MeOH glassy matrix at 77 K (c = 10^{-5} M), including the residuals (λ_{ex} = 376 nm, λ_{em} = 600 nm). Right: Fitting parameters including pre-exponential factors and confidence limits.

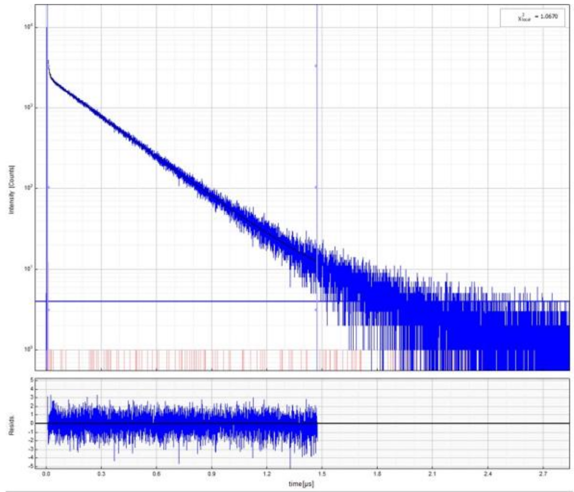

Parameter	Value	Error
A_1[kCnts/Chnl]	2.4020	± 0.0081
τ_1[ns]	259.19	± 0.66
I_1[kCnts]	2431.9	± 2.9
$A_{Rel\,1}$[%]	52.35	± 0.56
$I_{Rel\,1}$[%]	97.604	± 0.064
A_2[kCnts/Chnl]	2.187	± 0.053
τ_2[ns]	6.99	± 0.32
I_2[kCnts]	59.7	± 1.6
$A_{Rel\,2}$[%]	47.65	± 0.56
$I_{Rel\,2}$[%]	2.396	± 0.064
$Bkgr_{Dec}$[kCnts]	0.0040	± 0.0004
$\tau_{Av\,Int}$[ns]	253.15	± 0.53
$\tau_{Av\,Amp}$[ns]	139.0	± 1.6

Figure S21. Left: Time-resolved photoluminescence of [Pt(dba)(CN*t*Bu)] in an air-equilibrated CH_2Cl_2 solution at 298 K (c = 10^{-5} M), including the residuals (λ_{ex} = 376 nm, λ_{em} = 600 nm). Right: Fitting parameters including pre-exponential factors and confidence limits.

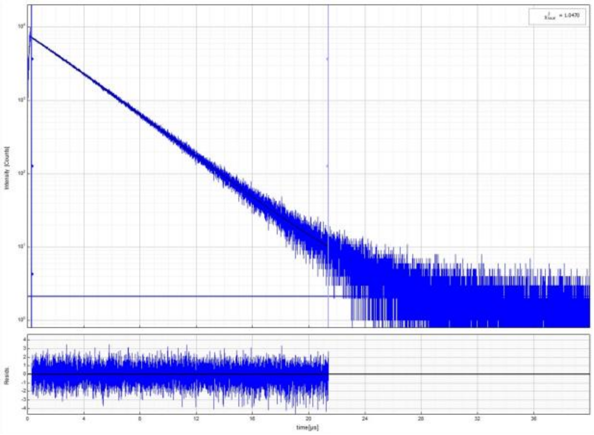

Parameter	Value	Error
A_1[kCnts/Chnl]	7.3483	± 0.0080
τ_1[ns]	3167.7	± 1.7
I_1[kCnts]	11638.5	± 9.0
$A_{Rel\,1}$[%]	100.0000	± 0.0000
$I_{Rel\,1}$[%]	100.0000	± 0.0000
$Bkgr_{Dec}$[kCnts]	-0.0008	± 0.0002
$\tau_{Av\,Int}$[ns]	3167.7	± 1.7
$\tau_{Av\,Amp}$[ns]	3167.7	± 1.7

Figure S22. Left: Time-resolved photoluminescence of [Pt(dba)(CN*t*Bu)] in an Ar-purged CH_2Cl_2 solution at 298 K (c = 10^{-5} M), including the residuals (λ_{ex} = 376 nm, λ_{em} = 600 nm). Right: Fitting parameters including pre-exponential factors and confidence limits.

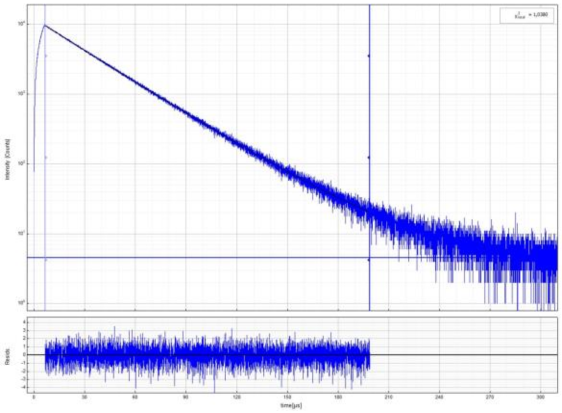

Parameter	Value	Error
A_1[kCnts/Chnl]	1,313	± 0,054
τ_1[ns]	38447	± 598
I_1[kCnts]	1577	± 48
$A_{Rel\,1}$[%]	13,67	± 0,56
$I_{Rel\,1}$[%]	18,35	± 0,55
A_2[kCnts/Chnl]	8,293	± 0,055
τ_2[ns]	27082	± 120
I_2[kCnts]	7018	± 44
$A_{Rel\,2}$[%]	86,33	± 0,56
$I_{Rel\,2}$[%]	81,65	± 0,55
$Bkgr_{Dec}$[kCnts]	0,0045	± 0,0007
$\tau_{Av\,Int}$[ns]	29167	± 54
$\tau_{Av\,Amp}$[ns]	28635	± 16

Figure S23. Left: Time-resolved photoluminescence of [Pt(dba)(CN*t*Bu)] in a frozen CH_2Cl_2/MeOH glassy matrix at 77 K (c = 10^{-5} M), including the residuals (λ_{ex} = 376 nm, λ_{em} = 600 nm). Right: Fitting parameters including pre-exponential factors and confidence limits.

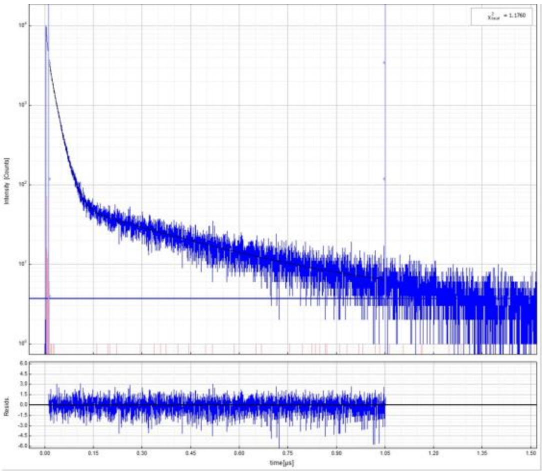

Parameter	Value	Error
A_1[kCnts/Chnl]	0.0601	± 0.0023
τ_1[ns]	335	± 24
I_1[kCnts]	78.7	± 3.3
$A_{Rel\,1}$[%]	1.500	± 0.053
$I_{Rel\,1}$[%]	22.41	± 0.70
A_2[kCnts/Chnl]	0.95	± 0.37
τ_2[ns]	7.3	± 2.7
I_2[kCnts]	27	± 24
$A_{Rel\,2}$[%]	23.7	± 9.7
$I_{Rel\,2}$[%]	7.7	± 6.6
A_3[kCnts/Chnl]	3.00	± 0.41
τ_3[ns]	21.0	± 1.2
I_3[kCnts]	245	± 22
$A_{Rel\,3}$[%]	74.8	± 9.7
$I_{Rel\,3}$[%]	69.9	± 6.5
$Bkgr_{Dec}$[kCnts]	0.0037	± 0.0008
$\tau_{Av\,Int}$[ns]	90.2	± 7.4
$\tau_{Av\,Amp}$[ns]	22.42	± 0.55

Figure S24. Left: Time-resolved photoluminescence of [Pt(dba)(Me_2Imd)] in an air-equilibrated CH_2Cl_2 solution at 298 K (c = 10^{-5} M), including the residuals (λ_{ex} = 376 nm, λ_{em} = 600 nm). Right: Fitting parameters including pre-exponential factors and confidence limits.

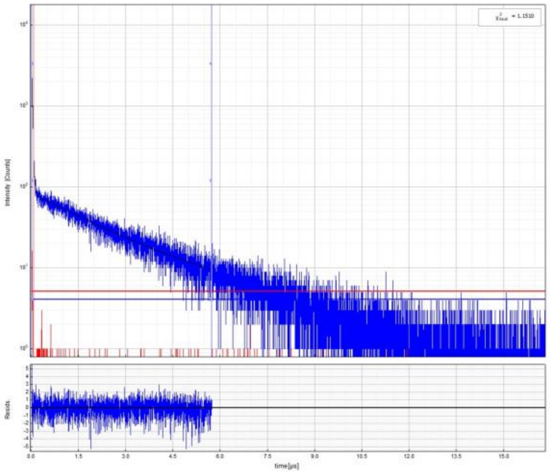

Parameter	Value	Error
A_1[kCnts/Chnl]	0.064	± 0.016
τ_1[ns]	2131	± 74
I_1[kCnts]	68	± 16
$A_{Rel\,1}$[%]	1.84	± 0.13
$I_{Rel\,1}$[%]	64.5	± 1.2
A_2[kCnts/Chnl]	3.39	± 0.54
τ_2[ns]	21.92	± 0.89
I_2[kCnts]	37.2	± 7.0
$A_{Rel\,2}$[%]	98.16	± 0.13
$I_{Rel\,2}$[%]	35.5	± 1.2
$Bkgr_{Dec}$[kCnts]	0.0041	± 0.0007
$Bkgr_{IRF}$[Cnts/Chnl]	5.1	± 2.0
$Shift_{IRF}$[ps]	1579	± 810
A_{Scat}[kCnts]	176	± 228
$\tau_{Av\,Int}$[ns]	1383	± 73
$\tau_{Av\,Amp}$[ns]	60.7	± 3.8

Figure S25. Left: Time-resolved photoluminescence of [Pt(dba)(Me_2Imd)] in an Ar-purged CH_2Cl_2 solution at 298 K (c = 10^{-5} M), including the residuals (λ_{ex} = 376 nm, λ_{em} = 600 nm). Right: Fitting parameters including pre-exponential factors and confidence limits.

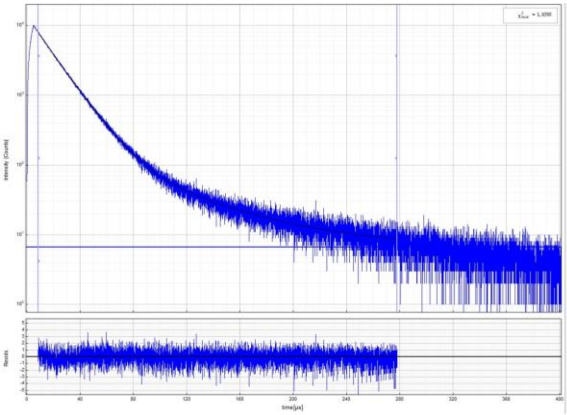

Parameter	Value	Error
A_1[kCnts/Chnl]	0,194	± 0,013
τ_1[ns]	59505	± 2241
I_1[kCnts]	360	± 11
$A_{Rel\,1}$[%]	2,45	± 0,16
$I_{Rel\,1}$[%]	8,74	± 0,27
A_2[kCnts/Chnl]	7,724	± 0,014
τ_2[ns]	15589	± 51
I_2[kCnts]	3763	± 14
$A_{Rel\,2}$[%]	97,55	± 0,16
$I_{Rel\,2}$[%]	91,26	± 0,27
$Bkgr_{Dec}$[kCnts]	0,0066	± 0,0005
$\tau_{Av\,Int}$[ns]	19426	± 145
$\tau_{Av\,Amp}$[ns]	16663	± 36

Figure S26. Left: Time-resolved photoluminescence of [Pt(dba)(Me_2Imd)] in a frozen CH_2Cl_2/MeOH glassy matrix at 77 K (c = 10^{-5} M), including the residuals (λ_{ex} = 376 nm, λ_{em} = 600 nm). Right: Fitting parameters including pre-exponential factors and confidence limits.

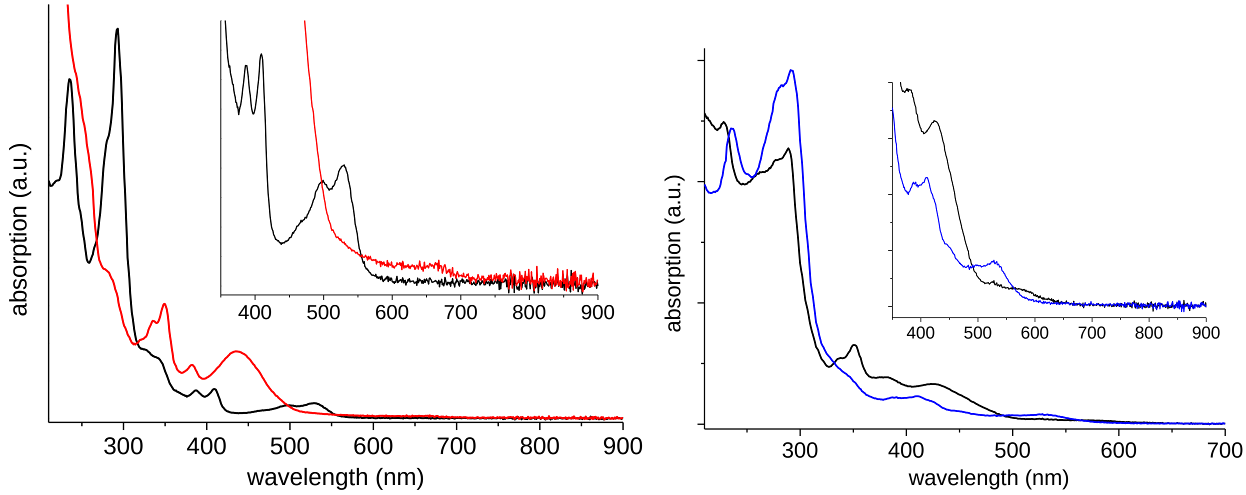

Figure S27. UV-vis absorption spectra of [Pt(dba)(dmso)] in THF/*n*-Bu_4NPF_6, recorded before (black trace) and after cathodic reduction (red trace) (left) and before (black trace) and after anodic oxidation (blue trace).

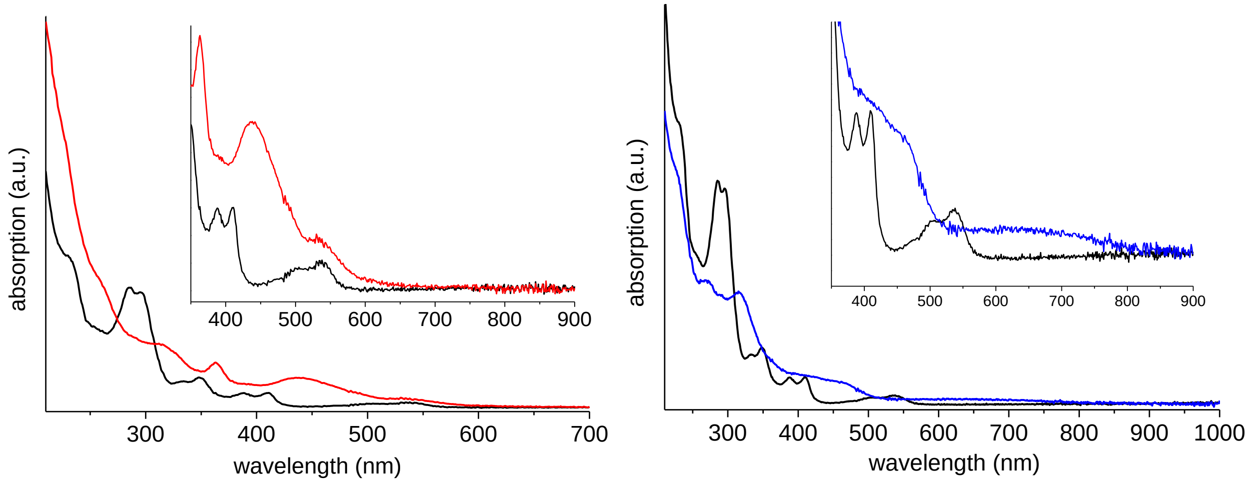

Figure S28. UV-vis absorption spectra of [Pt(dba)(PPh_3)] in THF/*n*-Bu_4NPF_6, recorded before (black trace) and after cathodic reduction (red trace) (left) and before (black trace) and after anodic oxidation (blue trace).

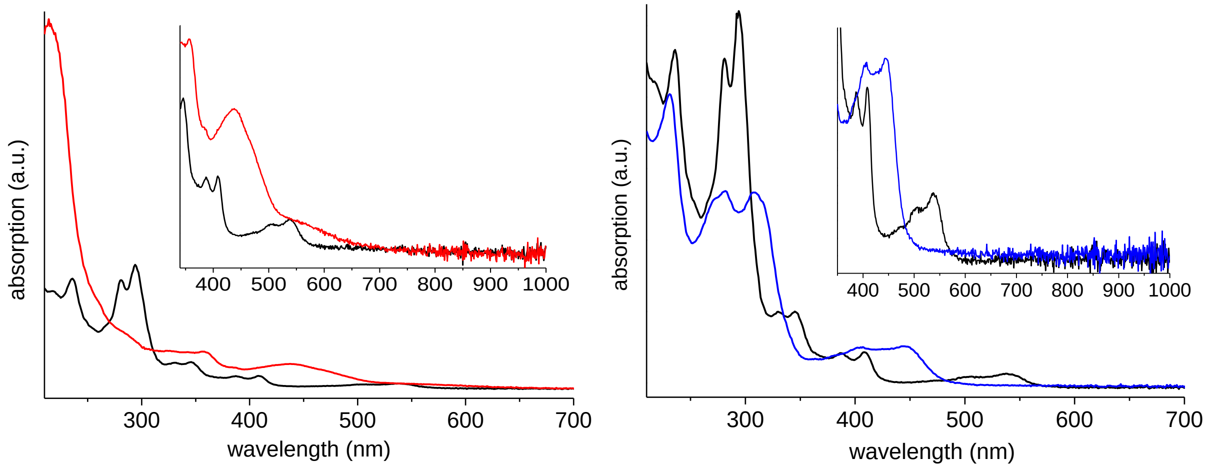

Figure S29. UV-vis absorption spectra of [Pt(dba)(CN*t*Bu)] in THF/*n*-Bu_4NPF_6, recorded before (black trace) and after cathodic reduction (red trace) (left) and before (black trace) and after anodic oxidation (blue trace) (right).

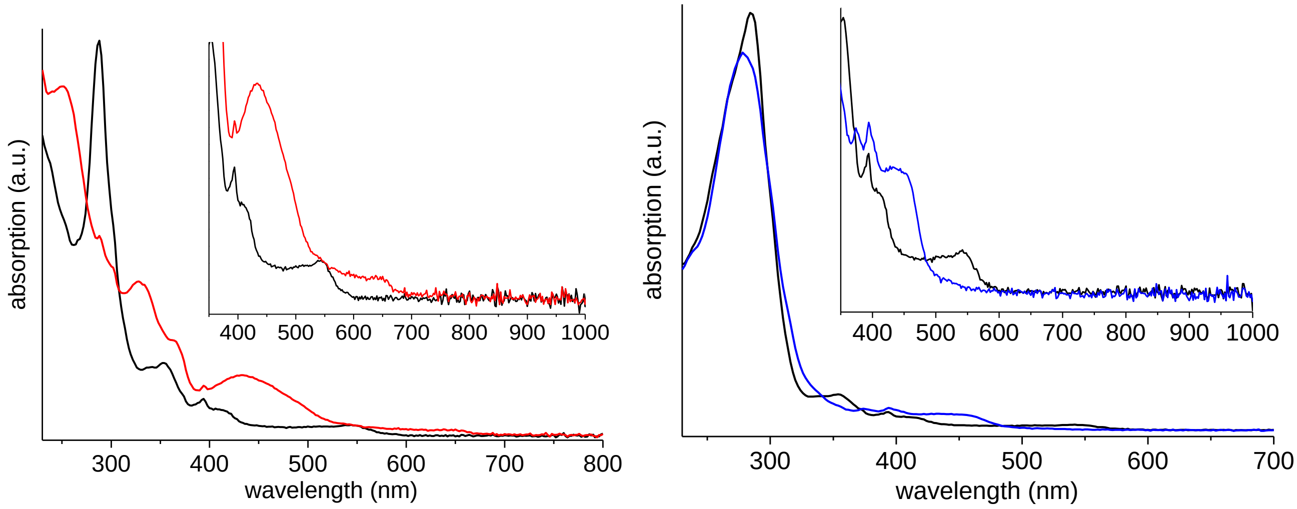

Figure S30. UV-vis absorption spectra of [Pt(dba)(Me_2Imd)] in THF/*n*-Bu_4NPF_6, recorded before (black trace) and after cathodic reduction (red trace) (left) and before (black trace) and after anodic oxidation (blue trace) (right).

Supplementary Tables

Table S1. Selected structure solution and refinement data for [Pt(dba)(dmso)], [Pt(dba)(PPh_3)], [Pt(dba)(CN*t*Bu)], and [Pt(dba)(Me_2Imd)].[a]

	[Pt(dba)(dmso)] [b]	[Pt(dba)(PPh_3)]	[Pt(dba)(CN*t*Bu)]	[Pt(dba)(Me_2Imd)]
formula	$C_{23}H_{17}NOSPt$	$C_{39}H_{26}NPPt$	$C_{26}H_{20}N_2Pt$	$C_{26}H_{19}N_3Pt$
formula wt (g/mol)	550.53	734.67	555.53	568.53
T (K)	100(2)	100(2)	100(2)	170(2)
crystal system	monoclinic	monoclinic	orthorhombic	orthorhombic
space group	*P* 2_1/c	*C* 2/c	*P* bca	*P* bca
cell a (Å)	10.5597(9)	26.920(5)	12.8708(8)	9.0642(3)
b (Å)	15.5689(12)	10.536(2)	13.5052(9)	20.6156(7)
c (Å)	10.9687(9)	26.047(5)	22.8678(15)	21.6365(10)
α (°)	90	90	90	90
β (°)	100.759(3)	118.15(3)	90	90
γ (°)	90	90	90	90
volume ($Å^3$) / Z	1771.6(3) / 4	6513.8(3) / 8	3974.9(4) / 8	4043.1(3) / 8
density calc. (g/cm^3)	2.064	1.498	1.857	1.868
abs. coefficient (cm^{-1})	8.05	2.386	7.074	6.959
F(000)	1056	2880	2144	2192
θ range data coll. (°)	2.3 to 33.1	1.354 to 19.738	2.360 to 35.031	1.882 to 26.945
index ranges	$-15 \le h \le 15$, $-22 \le k \le 22$, $-15 \le l \le 15$	$-32 \le h \le 32$, $-12 \le k \le 12$, $-29 \le l \le 30$	$-20 \le h \le 20$, $-20 \le k \le 21$, $-36 \le l \le 36$	$-11 \le h \le 11$, $-26 \le k \le 25$, $-27 \le l \le 27$
reflections collected	44234	20986	71762	34523
independent	5375 (R_{int}: 0.0489,	5775 (R_{int}: 0.0300,	8750 (R_{int}: 0.0450,	4322 (R_{int}: 0.1103,

reflections	R_{sigma}: 0.0292)	R_{sigma}: 0.0278)	R_{sigma}: 0.0263)	R_{sigma}: 0.0569)
completeness to θ	100%	100%	100%	100%
data / restr. / param.	5375 / 0 / 247	5775 / 0 / 380	8750 / 0 / 266	4322 / 0 / 274
goodness-of-fit on F^2	1.085	1.089	1.369	1.001
final R indices [I>2σ(I)]	R_1: 0.0236, wR_2: 0.0574	R_1: 0.0285, wR_2: 0.0725	R_1: 0.0316, wR_2: 0.0664	R_1: 0.0463, wR_2: 0.1139
R indices (all data)	R_1: 0.0260, wR_2: 0.587	R_1: 0.0330, wR_2: 0.0742	R_1: 0.0406, wR_2: 0.0692	R_1: 0.0921, wR_2: 0.1418
extinction coefficient	0.00084(13)	0.00086(5)	0.00024(4)	0.0008(1)
larg. diff. peak & hole	2.83/-1.58	1.05/–2.26	3.22/–2.55	1.65/–3.4
CCDC	2141888	2070867	2070866	2070868

[a] From single crystal X-ray diffraction using Mo-Ka (λ = 0.71073 Å) radiation for all except [Pt(dba)(PPh_3)] and synchrotron (λ = 0.56076 Å) radiation for [Pt(dba)(PPh_3)]. Least square refinement against $|F|^2$. [b] The structure of [Pt(dba)(dmso)] was alternatively determined at 298(2) K with the same space group and very similar further parameters; CCDC: 2141889.

Table S2. Selected experimental structural data of [Pt(dba)(dmso)], [Pt(dba)(PPh_3)], [Pt(dba)(CN*t*Bu)], and [Pt(dba)(Me_2Imd)].[a]

distances / Å	[Pt(dba)(dmso)]	[Pt(dba)(PPh_3)]	[Pt(dba)(CN*t*Bu)]	[Pt(dba)(Me_2Imd)]
Pt–N1	1.986(2)	2.003(4)	1.988(2)	1.999(9)
Pt–C1	2.128(3)	2.105(5)	2.085(3)	2.115(1)
Pt–C18	2.093(2)	2.099(5)	2.093(3)	2.086(1)
Pt–X	2.191(1)	2.230(1)	1.898(3)	1.986(1)
N1–C13	1.342(3)	1.337(7)	1.334(4)	1.337(1)
N1–C10	1.342(3)	1.333(5)	1.337(4)	1.352(1)
C13–C17	1.424(3)	1.440(6)	1.425(4)	1.393(1)
C10–C6	1.423(3)	1.420(6)	1.429(4)	1.422(1)
C17–C18	1.428(3)	1.431(7)	1.435(4)	1.442(1)
C6–C1	1.436(4)	1.439(6)	1.431(4)	1.427(1)
angles / °				
C1–Pt–C18	158.87(9)	158.6(2)	159.6(1)	159.1(4)
X–Pt–N1	173.18(7)	176.2(1)	177.6(1)	177.8(4)
N1–Pt–C1	79.1(1)	79.2(2)	80.1(1)	79.8(4)
N1–Pt–C18	79.92(9)	79.5(2)	79.5(1)	79.3(4)
C1–Pt–X	106.62(8)	104.5(1)	97.5(1)	101.9 (4)
C18–Pt–X	94.47(7)	96.8(1)	102.9(1)	98.9(4)
dihedral angles				
C1–Pt–X–Y	129.9(1)	–12.1(2)	123.1(1)	123.2(10)
C18–Pt–X–Y	–51.4(1)	169.7(2)	92.09(5)	–57.9(10)
N1–Pt–X–Y	–17.0(6)	–174.4(2)	163.3(5)	-93.2(10)
N1–C10–C6–C5	176.3(2)	-178.9(4)	–2.0(1)	178.8(9)
N1–C13–C17–C16	–179.4(2)	177.5(4)	0.8(1)	179.9(9)
N1–C10–C9–C8	–177.7(2)	–179.9(4)	–178.9(9)	–178.9(9)
C14–C12–C11–C9	–179.5(3)	–177.5(5)	–178.8(1)	–178.8(11)
Sum of ∧ around Pt	360.1	360.0	360.0	359.9

[a] From single crystal X-ray diffraction, see Table S1.

Table S3. Electrochemical data of the Pt(II) complexes.[a]

[Pt(dba)(L)]	Red			Ox	gap
	$E_{1/2}$ Red2	$E_{1/2}$ Red1	ΔRed1 –Red2	E_{pa} Ox1	ΔOx1– Red1
L = Me_2Imd	–3.02 (irr)	–2.10	0.92	0.55	2.65
L = PPh_3	–2.70 (irr)	–1.96	0.74	0.75	2.71
L = CN*t*Bu	–3.26	–1.89	1.37	0.80	2.69
L = dmso	–2.70 (irr)	–1.98 (irr)	0.72	0.93	2.91
[Pt(db(ph)a)(dmso)]	–2.52 (irr)	–1.89	0.63	1.00	2.89

[a] From cyclic voltammetry in *n*Bu_4NPF_6/THF (tetrahydrofuran). Potentials in V vs. ferrocene/ferrocenium; half-wave potentials ($E_{1/2}$) for reversible processes; cathodic peak potentials (E_{pc}) for irreversible reductions (irr); accuracy of potentials: ± 0.003 V.

Table S4. Experimental long-wavelength absorption maxima of the Pt(II) complexes.[a]

[Pt(dba)(L)]									
L =	λ_1(ε)	λ_2 (ε)	λ_3 (ε)	λ_4(ε)	λ_5 (ε)	λ_6 (ε)	λ_7 (ε)	λ_8 (ε)	cut-off
dmso	280 (32.0)	292 (47.8)	324 (10.5)	340 (8.3)	387 (3.7)	409 (4.1)	491 (1.7)	525 (1.9)	566
PPh_3	285 (50.1)	296 (50.2)	332 (11.6)	347 (12.7)	387 (6.0)	409 (6.2)	502 (1.6)	532 (2.1)	577
CN*t*Bu	280 (22.1)	294 (27.0)	326 (5.7)	343 (5.2)	387 (2.2)	408 (2.4)	501 (0.8)	533 (1.0)	568
Me_2Imd	287 (45.5)	300 (24.8)	338 (7.9)	352 (8.9)	393 (3.8)	406 (3.2)	507 (1.0)	536 (1.3)	580
[Pt(db(ph)a)(dmso)]		293 (39)		346 (6.8)				533 (1.4)	

[a] Measured in CH_2Cl_2. [b] Wavelength λ in nm, molar absorption coefficient ε (in 10^3 M^{-1} cm^{-1}), main maxima are underlined.

Table S5. Selected absorption maxima of the oxidized, parent, and reduced $[Pt(dba)(L)]^{+/0/-}$ complexes.[a]

L =	charge	λ_1	λ_2	λ_3	λ_4	λ_5	λ_6	λ_7	λ_8	λ_9	λ_{10}	λ_{11}
dmso	$[\ ]^+$	-	236	-	291	338		409	-	-	525	-
PPh_3	$[\ ]^+$	-	227	266	315	-	-	-	-	451	-	640
CN*t*Bu	$[\ ]^+$	-	231	281	307	-	-	405	-	446	-	-
Me_2Imd	$[\ ]^+$	-	-	278	-	374	-	395	432	-	-	
dmso	[ ]	-	235	292	-	340	387	409	-	496	529	-
PPh_3	[ ]	230	285	296	332	348	388	410	-	-	533	-
CN*t*Bu	[ ]	218	235	281	294	346	-	409	-	-	538	-
Me_2Imd	[ ]	-	-	287	-	353	-	394	-	-	545	
dmso	$[\ ]^-$	216	246	284	-	350	-	406	438	-	-	660
PPh_3	$[\ ]^-$	224	-	-	-	348	363	411	437		530	-
CN*t*Bu	$[\ ]^-$	214	-	280	325	355	-	-	436	-	-	570
Me_2Imd	$[\ ]^-$	-	250	-	327	-	-	394	431	-	545	640

[a] Measured in THF/*n*-Bu_4NPF_6, wavelength λ in nm, main maxima are underlined.

Part II

contents

Figure S31. 600 MHz ^{1}H NMR spectra of [Pt(dba)(PPh_3)] in CD_2Cl_2.
Figure S32. 600 MHz ^{31}P NMR spectrum of [Pt(dba)(PPh_3)] in CD_2Cl_2.
Figure S33. 600 MHz H,H COSY NMR spectrum of [Pt(dba)(PPh_3)] in CD_2Cl_2.
Figure S34. 600 MHz H,H NOESY NMR spectrum of [Pt(dba)(PPh_3)] in CD_2Cl_2.
Figure S35. 600 MHz ^{1}H NMR spectrum of [Pt(dba)(CN*t*Bu)] in CD_2Cl_2.
Figure S36. 300 MHz ^{13}C-135-DEPTQ-[^{1}H] NMR spectra of [Pt(dba)(CN*t*Bu)] in CD_2Cl_2.
Figure S37. 600 MHz H,H COSY NMR spectrum of [Pt(dba)(CN*t*Bu)] in CD_2Cl_2.
Figure S38. 600 MHz ^{1}H NMR spectrum of [Pt(dba)(Me_2Imd)] in CD_2Cl_2.
Figure S39. 300 MHz ^{13}C-135-DEPTQ-[^{1}H] NMR spectra of [Pt(dba)(Me_2Imd)] in CD_2Cl_2.
Figure S40. 600 MHz H,H COSY NMR spectrum of [Pt(dba)(Me_2Imd)] in CD_2Cl_2.
Figure S41. 300 MHz ^{1}H-^{195}Pt-HMBC NMR spectrum of [Pt(dba)(Me_2Imd)] in CD_2Cl_2.
Figure S42. ESI-MS(+) of [Pt(dba)(PPh_3)].
Figure S43. ESI-MS(+) of [Pt(dba)(CN*t*Bu)].
Figure S44. EI-MS(+) of [Pt(dba)(Me_2Imd)].

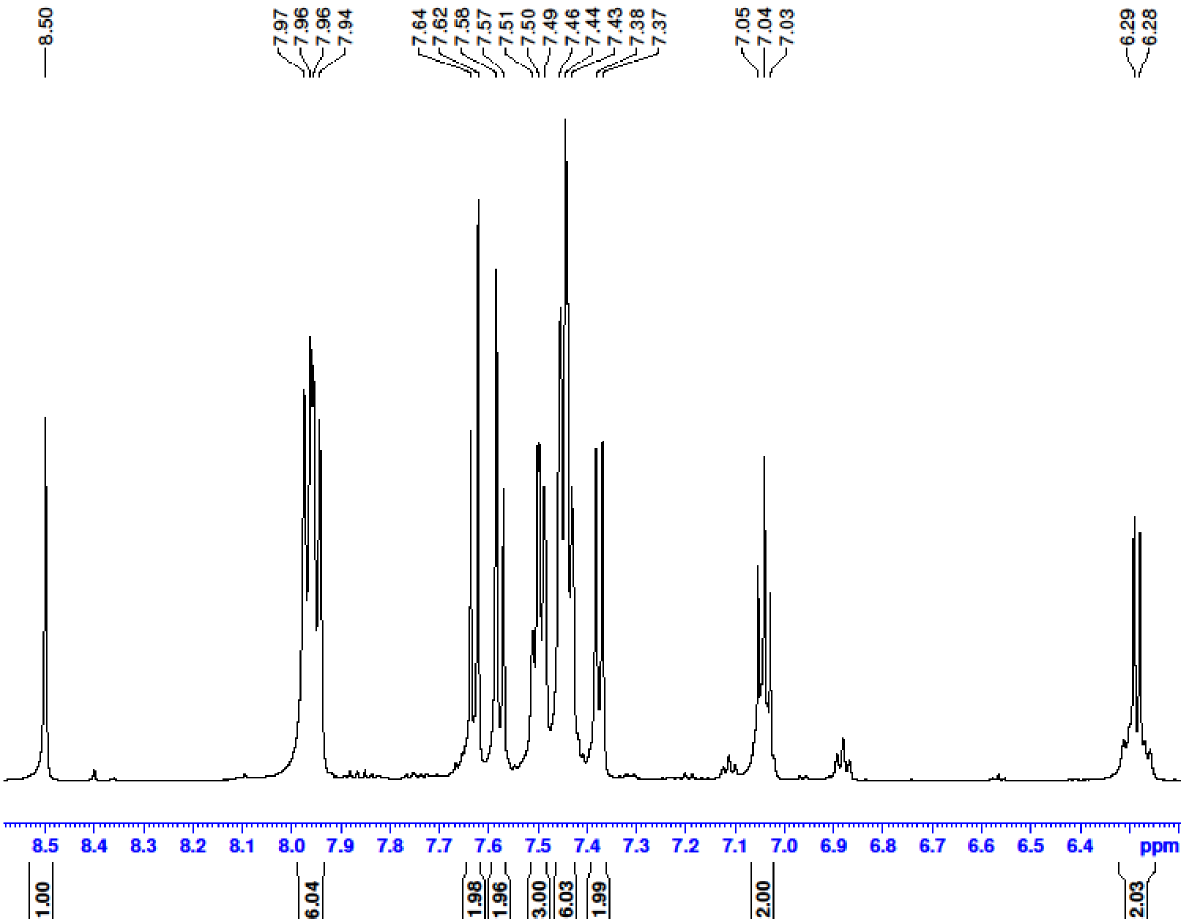

Figure S31. 600 MHz ^{1}H NMR spectrum of [Pt(dba)(PPh_3)] in CD_2Cl_2.

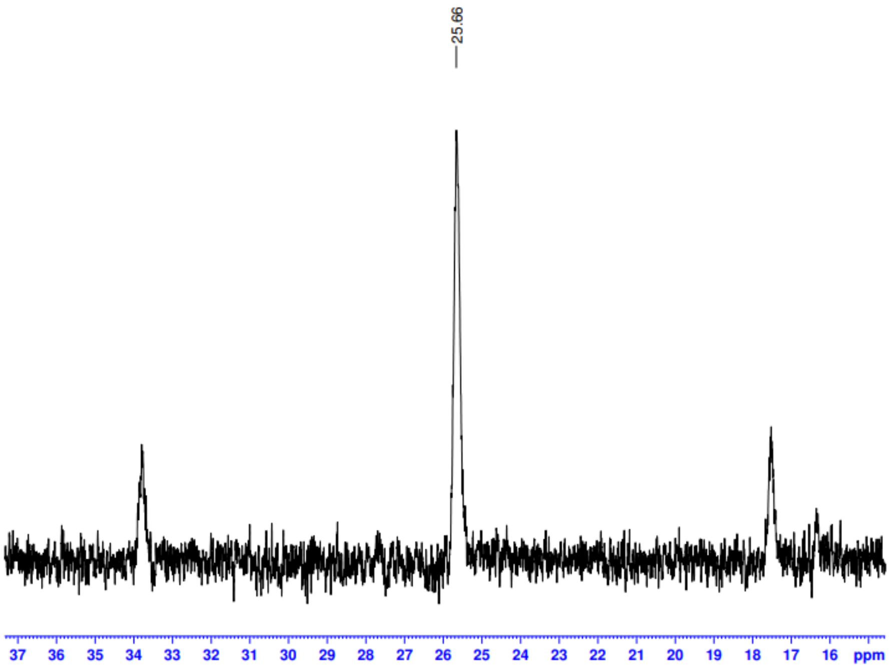

Figure S32 600 MHz ^{31}P NMR spectrum of [Pt(dba)(PPh_3)] in CH_2Cl_2.

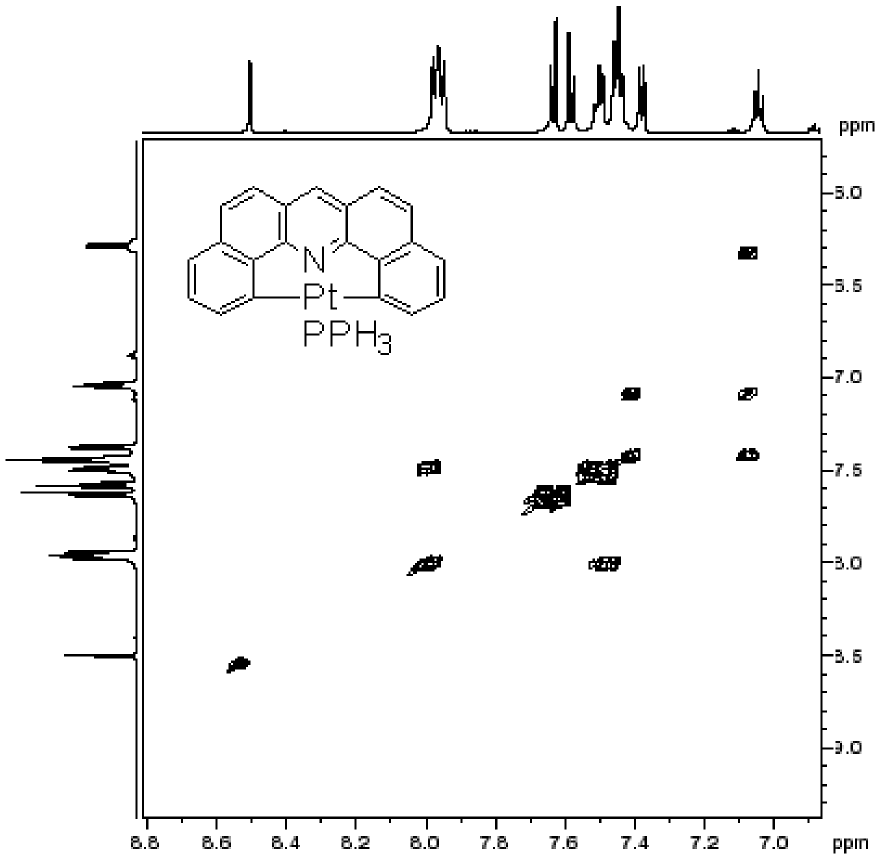

Figure S33. 600 MHz H,H COSY NMR spectrum of [Pt(dba)(PPh_3)] in CD_2Cl_2.

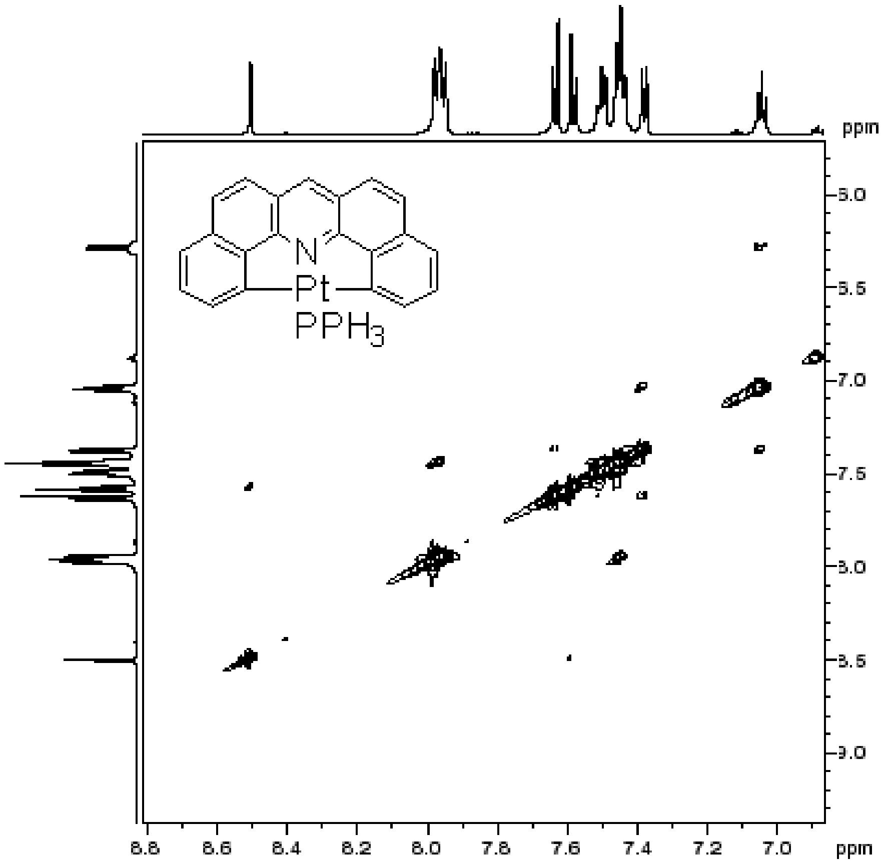

Figure S34. 600 MHz H,H NOESY NMR spectrum of [Pt(dba)(PPh_3)] in CH_2Cl_2.

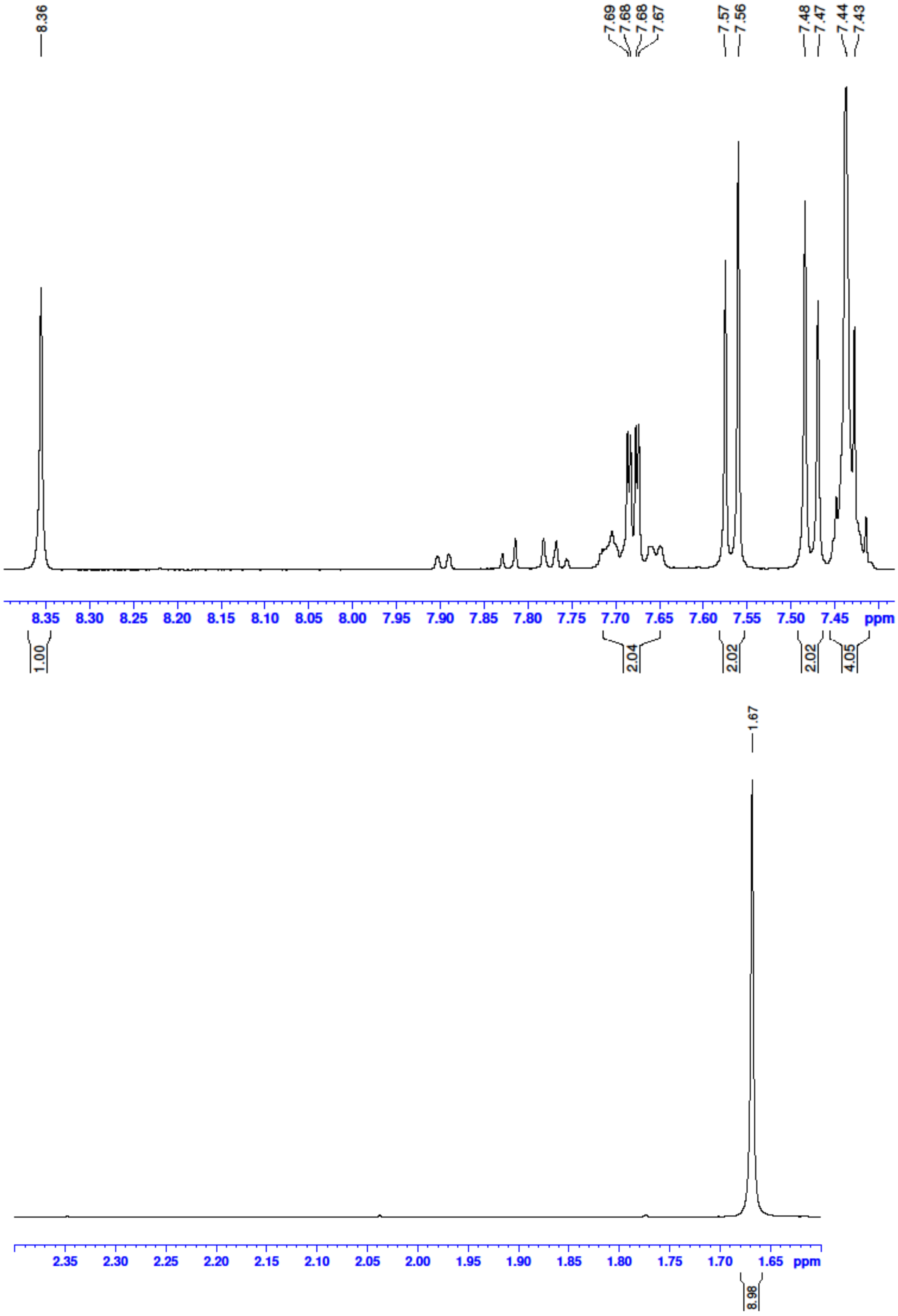

Figure S35. 600 MHz ^{1}H NMR spectra of [Pt(dba)(CN*t*Bu)] in CD_2Cl_2.

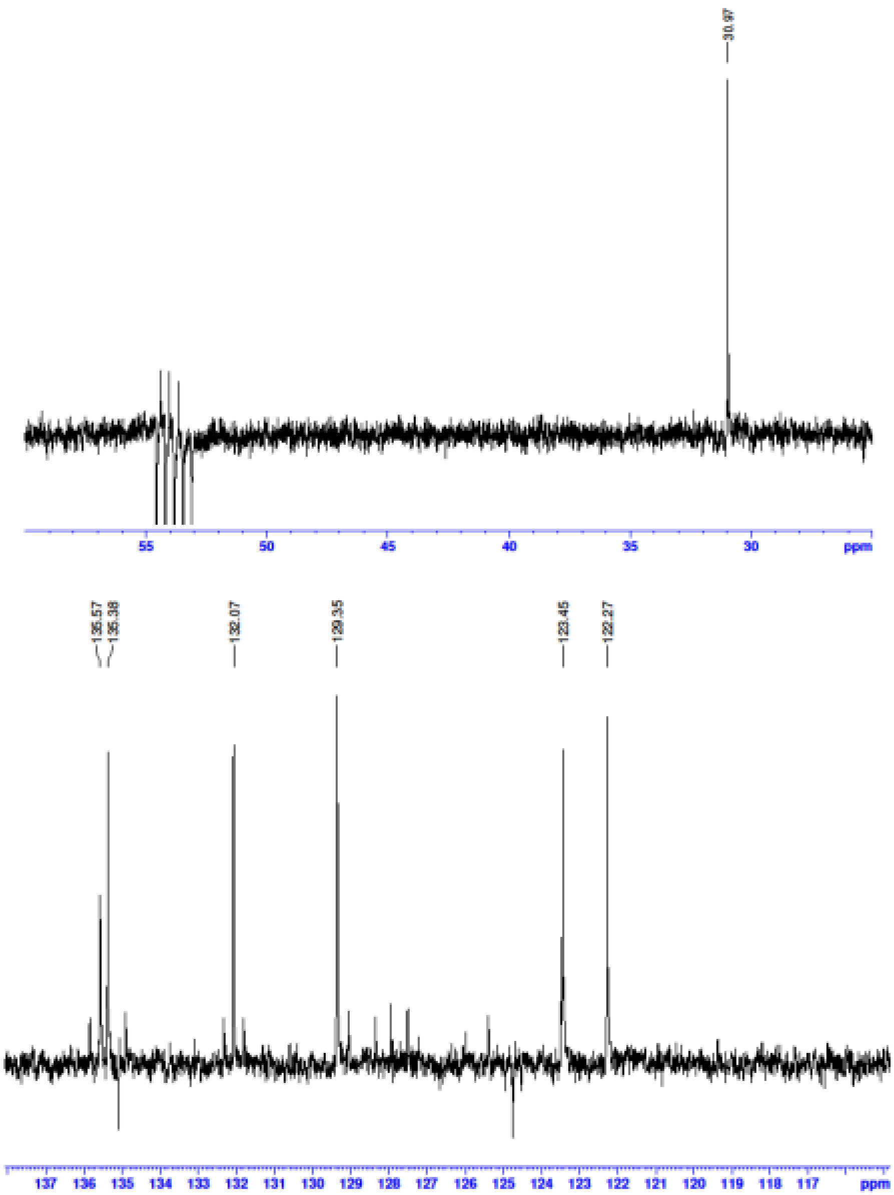

Figure S36. 300 MHz ^{13}C-135-DEPTQ-[^1H] NMR spectra of [Pt(dba)(CN*t*Bu)] in CD_2Cl_2.

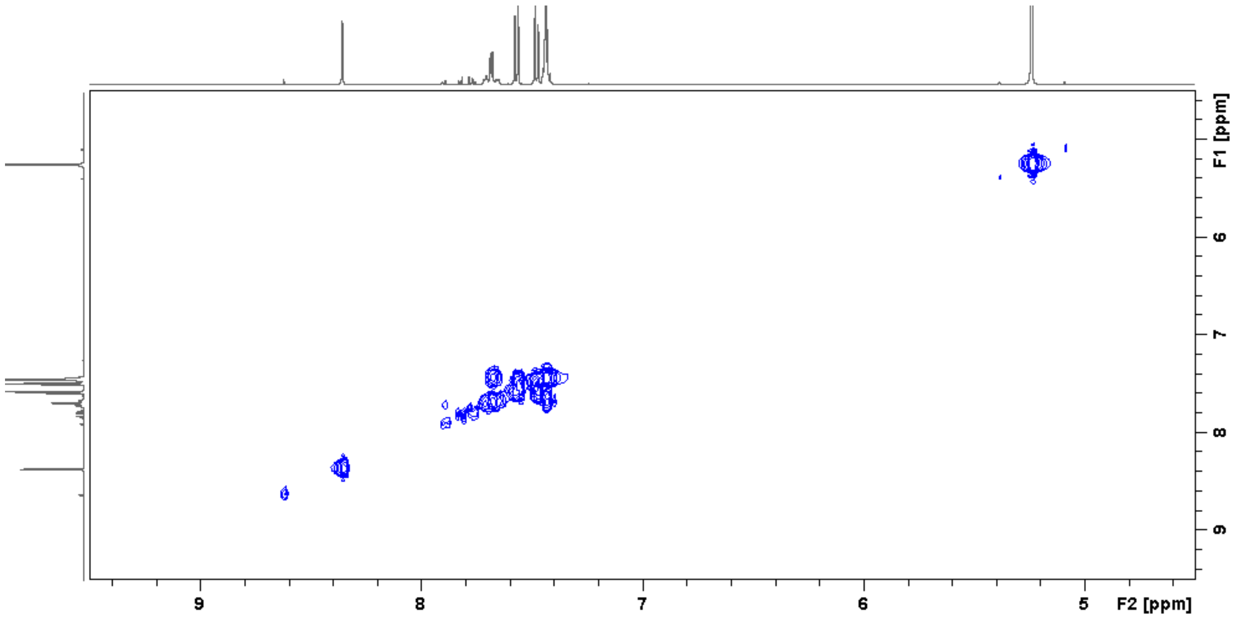

Figure S37. 600 MHz H,H COSY NMR of [Pt(dba)(CN*t*Bu)] in CD_2Cl_2.

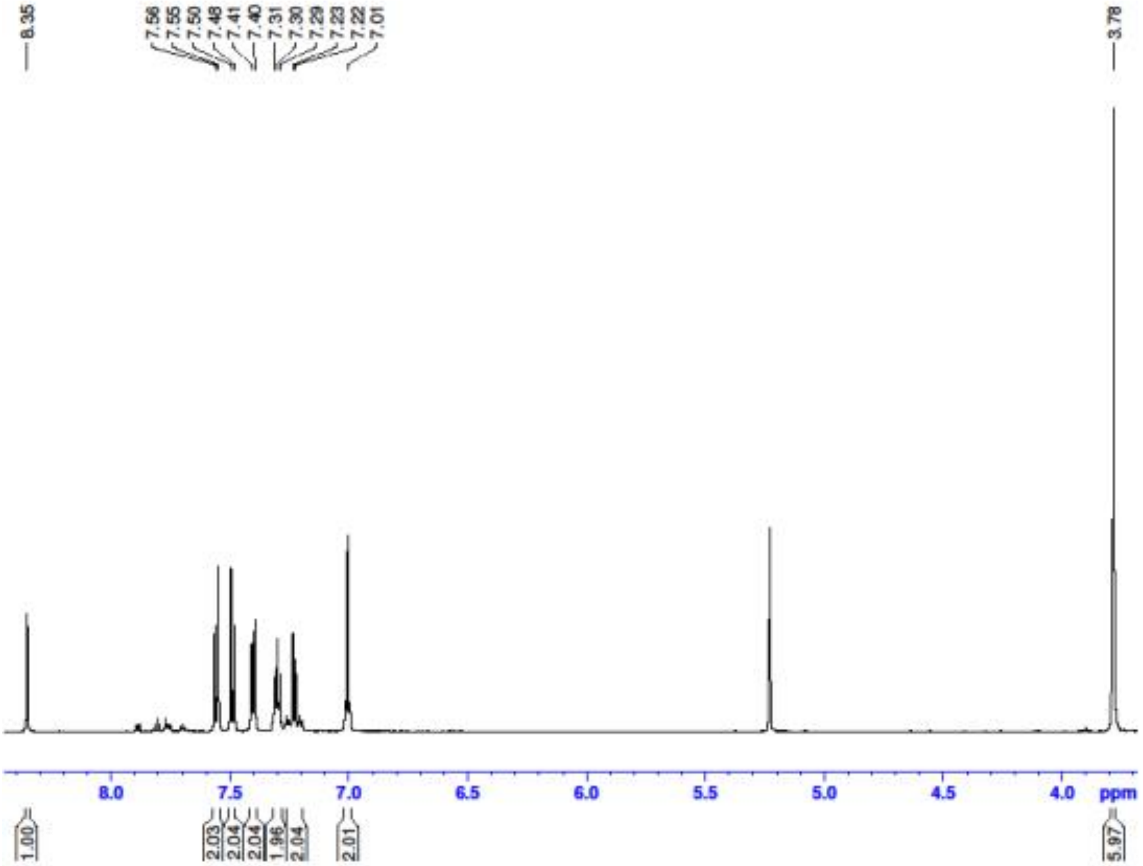

Figure S38. 600 MHz ^1H NMR spectrum of [Pt(dba)(Me_2Imd)] in CD_2Cl_2.

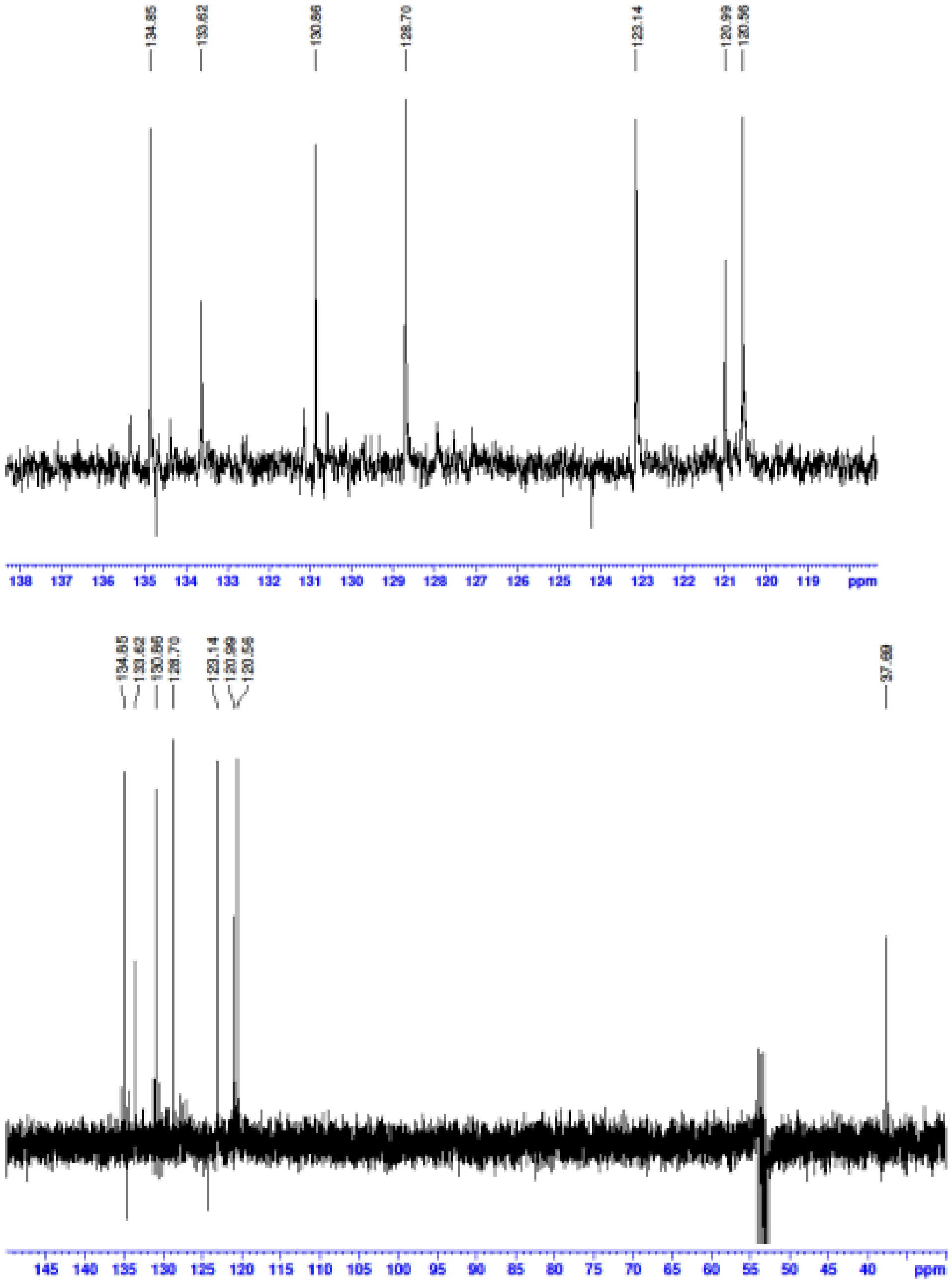

Figure S39. 300 MHz ^{13}C-135-DEPTQ-[^{1}H] NMR spectrum of [Pt(dba)(CN*t*Bu)] in CD_2Cl_2.

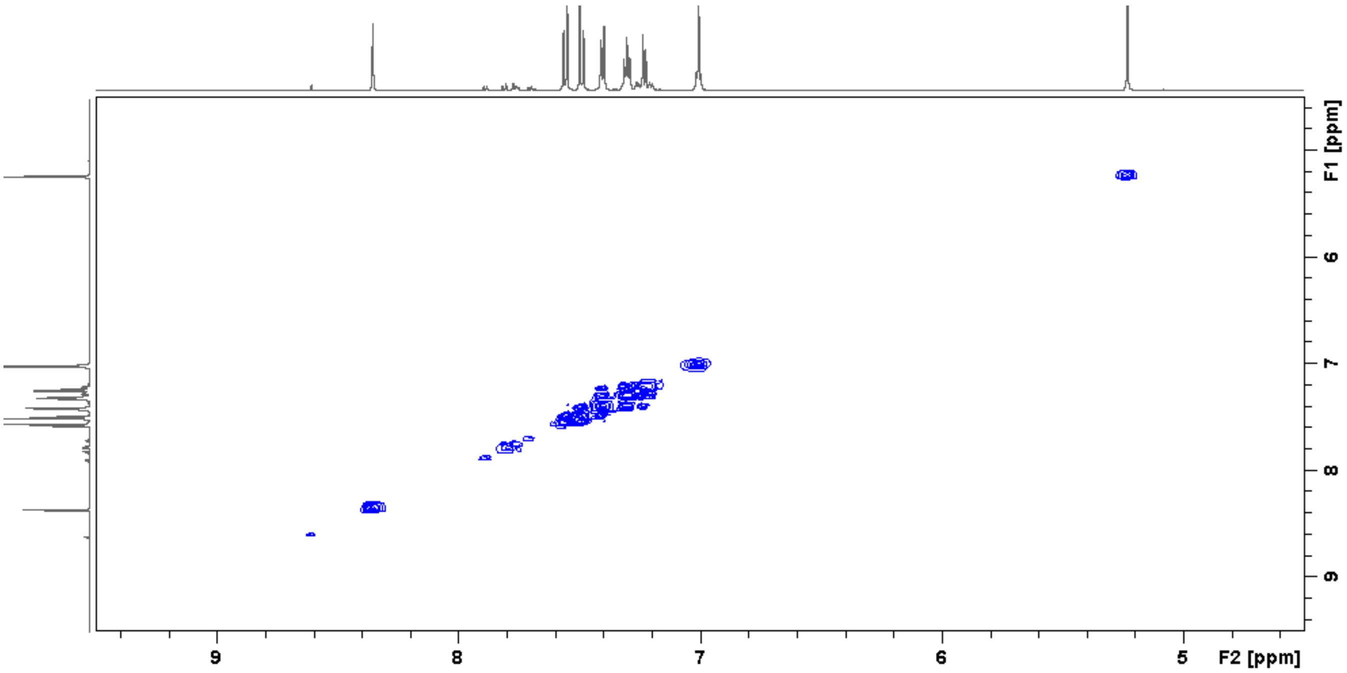

Figure S40. 600 MHz H,H COSY NMR of [Pt(dba)(Me_2Imd)] in CD_2Cl_2.

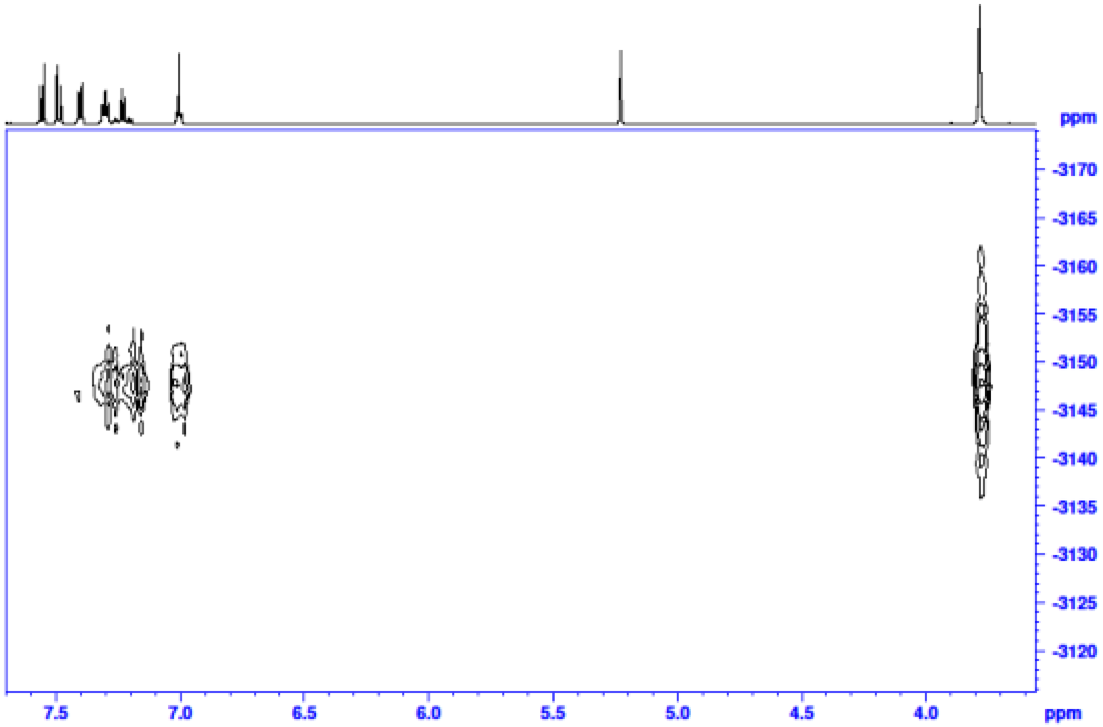

Figure S41. 300 MHz ^1H-^{195}Pt-HMBC NMR spectrum of [Pt(dba)(Me_2Imd)] in CD_2Cl_2.

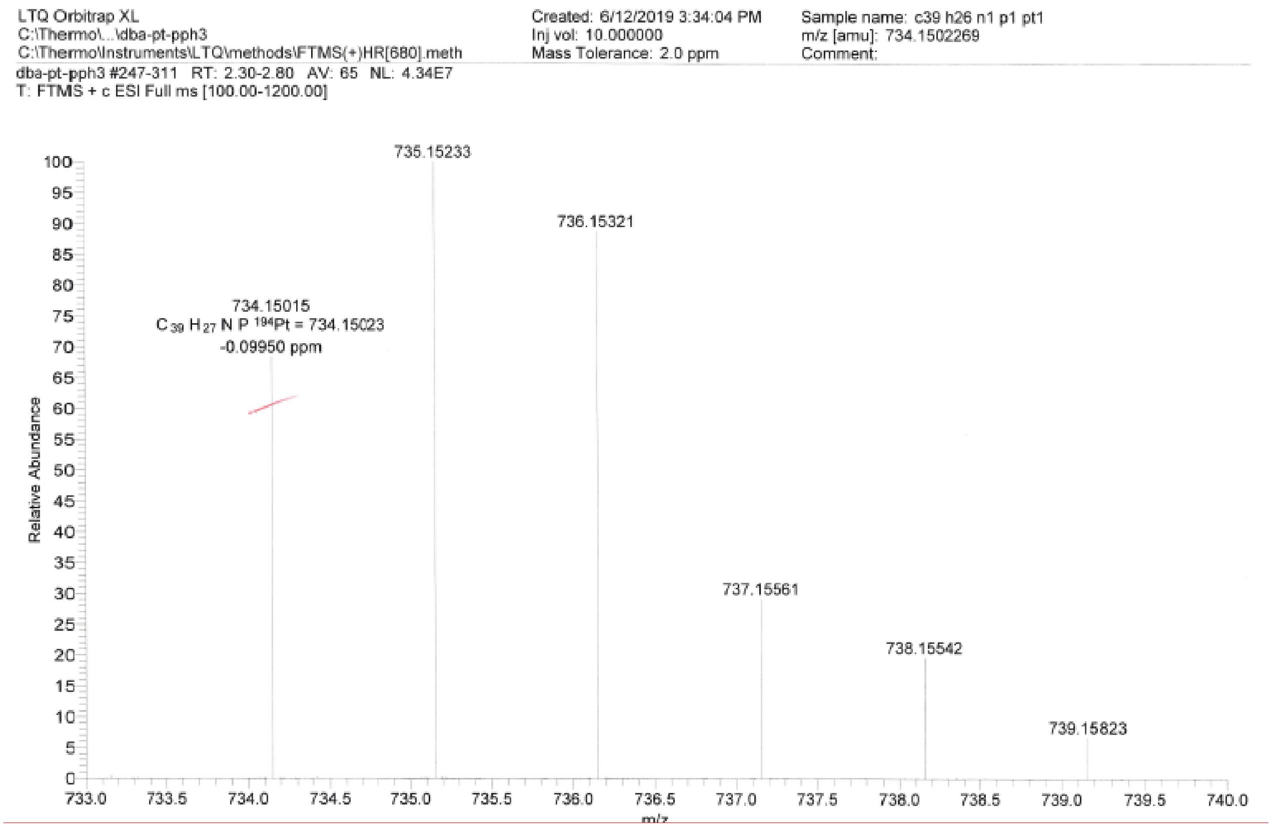

Figure S42. ESI-MS(+) of [Pt(dba)(PPh_3)].

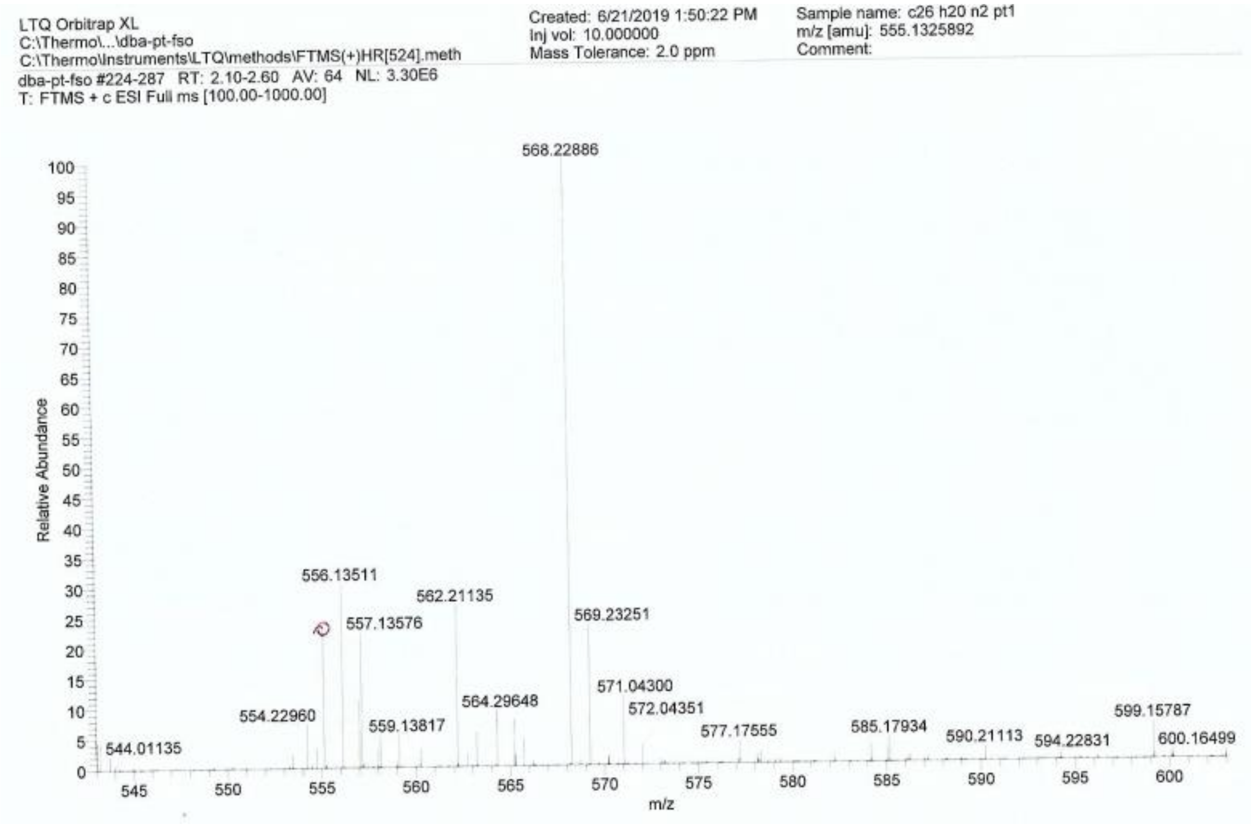

Figure S43. ESI-MS(+) of [Pt(dba)(CN*t*Bu)].

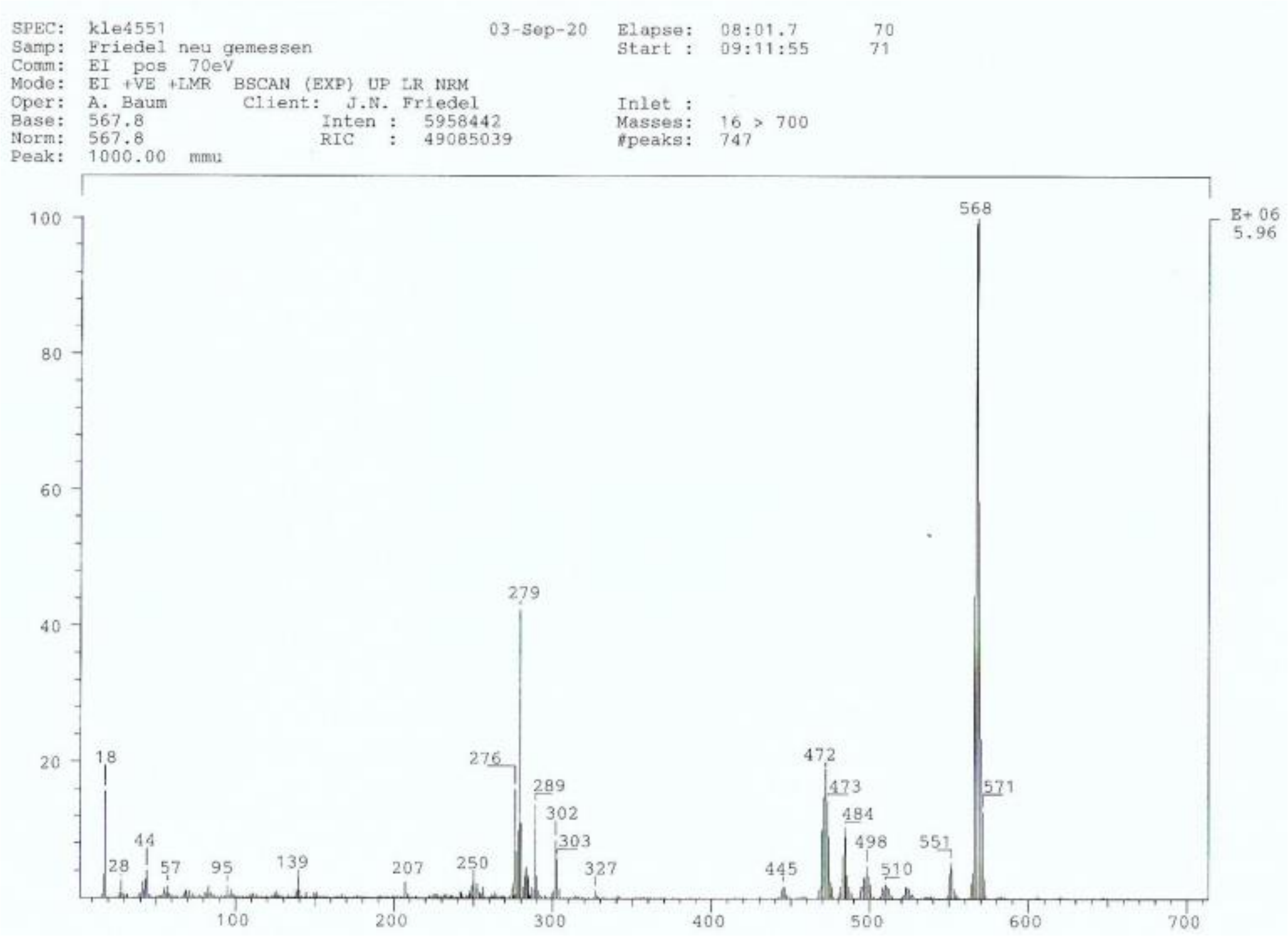

Figure S44. EI-MS(+) of [Pt(dba)(Me_2Imd)].
